# Radiolabeled *para*-I-nimesulide: an unexpected tracer for imaging peripheral inflammation

**DOI:** 10.3389/fnume.2025.1720380

**Published:** 2026-01-02

**Authors:** Yumi Yamamoto, Kentaro Imai, Yohei Saito, Fumihiko Yamamoto

**Affiliations:** Division of Radiopharmacy, Faculty of Pharmaceutical Sciences, Tohoku Medical and Pharmaceutical University, Sendai, Japan

**Keywords:** cyclooxygenase-2, imaging, nimesulide, brain, inflammation

## Abstract

**Introduction:**

In our previous studies, we demonstrated that nimesulide derivatives bearing iodine at the *para*-position of the phenyl ring exhibit potent inhibitory activity against cyclooxygenase-2 (COX-2). In the present study, we investigated whether radioiodinated derivatives of nimesulide could serve as COX-2 imaging agents for single-photon emission computed tomography (SPECT), with a particular focus on their potential to visualize COX-2 expression in the brain.

**Methods:**

^125^I-labeled derivatives substituted at the *para*- or *meta*-positions were synthesized from the corresponding tributyltin precursors with satisfactory radiochemical yields and purities. Biodistribution studies and *ex vivo* autoradiography in normal mice revealed that [^125^I]*para*-I nimesulide exhibited limited brain penetration and did not accurately reflect the distribution of COX-2 in the brain, suggesting it is unsuitable as a brain-targeted imaging agent.

**Results:**

In contrast, biodistribution and blocking experiments in a mouse model of inflammation demonstrated selective accumulation of [^125^I]*para*-I nimesulide in inflamed regions, which was significantly inhibited by COX-2–selective inhibitors. Moreover, [^125^I]*para*-I nimesulide exhibited high radiochemical purity and persistent *in vivo* stability, but strong plasma albumin binding likely restricted its brain uptake.

**Discussion:**

These findings indicate that while [^125^I]*para*-I nimesulide has limited potential for brain-targeted COX-2 imaging, it may serve as a promising tracer for detecting COX-2 expression in peripheral tissues. Importantly, this study also highlighted that the electronic properties of substituents strongly influence metabolic stability, providing valuable insights for the design of future COX-2–targeted molecular imaging agents.

## 1 Introduction

Cyclooxygenase-2 (COX-2) is an inducible enzyme that mediates prostaglandin synthesis and contributes to pathological processes, including inflammation, pain, and fever (1). While normally expressed at low levels, it is markedly upregulated by cytokines and growth factors. It is constitutively present in certain tissues, including the brain and kidneys, where it also supports physiological functions such as synaptic plasticity, memory, and regulation of cerebral blood flow (1, 2). In neurodegenerative diseases such as Alzheimer's and Parkinson's, COX-2 overexpression promotes inflammation, oxidative stress, and neuronal death. Additionally, it has been linked to acute conditions such as hypoxia, ischemia, and seizures (3–5). Given its diverse and critical roles, COX-2 has emerged as an important therapeutic target in the central nervous system. Against this background, molecular imaging approaches—particularly radiolabeled probes for positron emission tomography (PET) and single-photon emission computed tomography (SPECT)—offer valuable means to assess COX-2 activity *in vivo* (6, 7). Such imaging can aid in evaluating neuroinflammation and drug target occupancy; however, significant challenges remain, including blood–brain barrier (BBB) penetration, metabolic stability, and selectivity over COX-1 (6, 8), all of which are major obstacles to clinical application. While human studies are still limited, recent work with [^11^C]MC1 has suggested the feasibility of detecting low levels of COX-2 expression in the healthy brain, underscoring the importance of considering disease-specific differences in the timing and distribution of COX-2 induction (6, 9). Building on these findings, subsequent clinical and pre-clinical studies have further advanced COX-2 molecular imaging. In particular, [^11^C]MC1 has enabled quantification of low-level expression in humans with confirmation of tracer specificity through blocking studies (9), while other tracers—including [^11^C]TMI, [(^18^F)] Pyricoxib, and [^11^C]BRD1158—have been evaluated in pre-clinical models and shown varying strengths, such as favorable BBB penetration or selective target binding depending on the compound (10–13). Despite these advances, significant challenges remain for clinical translation, including sensitivity in low-expression regions, intersubject variability, and issues related to tracer pharmacokinetics and metabolic stability (9–13), underscoring a clear and unmet need for improved imaging agents.

**Figure 1:**
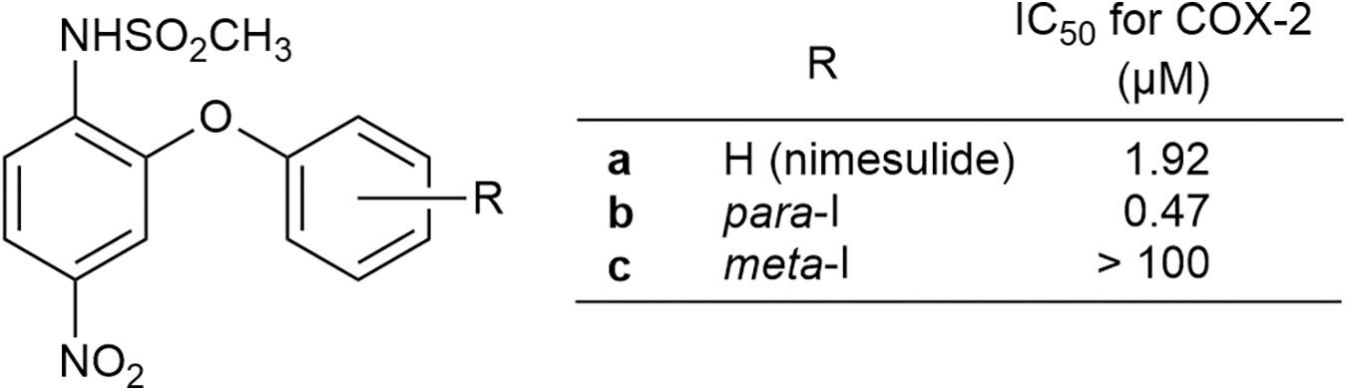
Chemical structures of nimesulide and nimesulide iodinated analogs. The IC₅₀ values shown in this figure were cited from reference (24).

Nimesulide, a COX-2-selective nonsteroidal anti-inflammatory drug (NSAID), has attracted attention as a particularly promising scaffold for molecular imaging probes due to its high COX-2 selectivity and favorable structural characteristics (Figure 1). Clinical studies have demonstrated that nimesulide can inhibit COX-2 activity while exerting minimal effects on COX-1. Molecular modeling has revealed that its selectivity is supported by subtle differences in the active sites of the COX isoforms (14–17). Furthermore, derivatives based on the nimesulide scaffold have been developed to enhance COX-2 selectivity or confer additional properties, such as antitumor activity, highlighting its structural versatility (18). In terms of radiolabeled imaging applications, [^11^C]nimesulide derivatives have been used to assess aromatase expression in breast cancer, and ^99m^Tc-labeled nimesulide has been employed to evaluate transdermal drug permeability (19, 20), suggesting the broad utility of this chemical framework.

In our previous studies, we designed and synthesized methoxy-substituted nimesulide derivatives as candidate COX-2 imaging agents targeting the brain and evaluated them *in vitro* and *in vivo* (21–23). In particular, the para-methoxy-nimesulide derivative, labeled with carbon-11 at the methoxy carbon, exhibited moderate COX-2 selective inhibitory activity (IC_50_ for COX-2 = 2.31 μM) and produced a small but significant specific signal *in vivo*. *Ex vivo* autoradiography further revealed heterogeneous brain distribution likely attributable to COX-2, indicating region-specific binding. However, critically, the brain uptake of this derivative in mice was low, suggesting limited BBB penetration, and it was rapidly metabolized *in vivo*. These findings highlight the key challenges for developing brain-targeted COX-2 imaging agents, namely the essential need for improving COX-2-specific binding, enhancing brain penetration, and increasing metabolic stability.

Building on these findings, the present study focused on nimesulide derivatives in which iodine was introduced at the *meta* or *para*-position of the nimesulide benzene ring (**1b** and **1c**) as a direct strategy to overcome the previously observed limitations. Both derivatives were stable *in vitro*, and the *para*-iodo derivative (**1b**) exhibited higher COX-2 selective inhibitory activity than the parent nimesulide scaffold (Figure 1) (23, 24). These properties suggest potential improvements in COX-2-specific binding and metabolic stability for the development of brain-targeted COX-2 imaging agents. Therefore, the present study aimed to evaluate whether the *para*-iodo-nimesulide derivative could serve as a superior SPECT imaging agent targeting COX-2 in the brain, with a focus on addressing the specific challenges of improving specific binding, brain penetration, and metabolic stability. For this purpose, both the *para*- and *meta*-iodo derivatives were labeled with iodine-125, and *in vivo* brain distribution and *ex vivo* autoradiography were first assessed for specific binding. Based on these results, further evaluations were conducted to explore the potential application in peripheral tissues, using an inflammation model in mice, along with assessments of *in vivo* metabolic stability and plasma protein binding.

## 2 Materials and methods

### 2.1 General

All chemicals, reagents, and solvents were of the highest purity available and were used without further purification. The progress of the reactions was monitored using TLC on silica gel 60 F254 glass plates (#1.05715.0001, Merck Millipore, Darmstadt, Germany), and the spots were visualized under UV light. Column chromatography was performed using silica gel 60 Å, 200–400 mesh (#288594, Sigma-Aldrich Inc., St. Louis, MO, USA). All melting points (mp) were determined on a Yanaco melting point apparatus (Yanagimoto Ind. Co., Kyoto, Japan) and were uncorrected. ^1^H-NMR spectra were recorded on a JNM-LA600 (JEOL Ltd., Tokyo, Japan) NMR spectrometer, and ^13^C-NMR spectra were recorded on a JNM-ECZ600R/S1 (JEOL Ltd.) NMR spectrometer. All NMR spectra were obtained using tetramethylsilane as an internal standard in dimethyl sulfoxide (DMSO)-d6, and the chemical shifts are reported in δ (ppm). Broadband proton-decoupled ^13^C-NMR measurements were performed to observe all carbon signals, including quaternary carbons. DEPT-90 and DEPT-135 experiments were used to distinguish CH, CH_2_, and CH_3_ groups: DEPT-90 detects only CH carbons, whereas DEPT-135 shows CH and CH_3_ as positive peaks and CH_2_ as negative peaks. Standard JEOL pulse programs were used for all ^13^C-NMR experiments. The IR spectra were recorded with a Spectrum One FT-IR Spectrometer (PerkinElmer, Inc., Waltham, MA, USA). Fast atom bombardment (FAB) mass spectra and high-resolution FAB mass spectra were recorded on a JMS-700 (JEOL Ltd.) using 3-nitrobenzyl alcohol (NBA) as the matrix.

Iodine-125 radionuclide was purchased from PerkinElmer, Inc. (now Revvity) as product NEZ033L. Radioactivity was quantified using the auto-well gamma counter (2470 WIZARD^2^; PerkinElmer, Inc.) and RI dose calibrator (CRC-55tR; Capintec, renamed Mirion Technologies, Inc., Atlanta, GA, USA).

HPLC was performed using a Waters liquid chromatography system (600 controller, 2487 dual λ absorbance detector; Milford, MA, USA) and a gamma survey meter (TCS172, Aloka Co., Ltd., Tokyo, Japan). UV and radioactivity signals from HPLC were collected and analyzed using the Chromato-Pro system (Runtime Instruments Co., Ltd., Tokyo, Japan).

Male BALB/cCrSlc mice (7–8 weeks old) were purchased from Japan SLC Inc. (Shizuoka, Japan) and acclimatized to the laboratory environment for at least 1 week prior to the experiments. Inhalation anesthesia with isoflurane was performed using a small-animal anesthesia system equipped with an excess gas scavenging function (WP-SAA01, LMS Co., Ltd., Tokyo, Japan). All animal experiments were approved by the Animal Care and Use Committee of Tohoku Medical and Pharmaceutical University and conducted in accordance with the university's animal care guidelines.

### 2.2 Chemistry

#### 2.2.1 *N*-(4-Nitro-2-phenoxy-phenyl)methanesulfonamide (1a), *N*-[2-(4-iodophenoxy)-4-nitrophenyl] methanesulfonamide (1b), *N*-[2-(3-iodophenoxy)-4-nitrophenyl] methanesulfonamide (1c)

Compounds **1a–c** were prepared as previously described (24).

#### 2.2.2 *N*-{4-Nitro-2-[4-(tributylstannyl) phenoxy] phenyl} methanesulfonamide (2b).

Tributyltin derivatization of **1b** was performed with modifications based on the reaction conditions reported in the literature (25), and the conditions were optimized experimentally. Under a nitrogen atmosphere, compound **1b** (20.6 mg, 0.047 mmol) was dissolved in dry toluene (8 mL), followed by the addition of tetrakis(triphenylphosphine)palladium(0) (2.8 mg, 2.4 μmol, 0.05 eq) and bis(tributyltin) (57.5 μL, 66.0 mg, 0.114 mmol, 2.42 eq). The mixture was heated at 120 °C for 4 h under reflux. The reaction mixture was allowed to cool, and a saturated aqueous solution of potassium fluoride was added. The mixture was then stirred at room temperature (maintained at approximately 25 °C) for 1 h. The reaction mixture was filtered through Celite, and the filtrate was subjected to liquid–liquid extraction using chloroform and water. The combined organic phase was dried and filtered, and the solvent was removed under reduced pressure. The residue was purified using chromatography on silica gel with chloroform:hexane:acetone in a 1:18:1 proportion, which yielded **2b** (14.0 mg, 49.9%) as a yellow solid: mp: 48–50 °C; ^1^H-NMR (600 MHz, DMSO-*d_6_*) δ ppm: 10.16 (*s*, 1H), 8.03 (*dd*, *J* = 2.6 Hz, 9.2 Hz, 1H), 7.72 (*d*, *J* = 8.8 Hz, 1H), 7.56 (*d*, *J* = 2.6 Hz, 1H), 7.51 (*d*, *J* = 8.4 Hz, 2H), 7.12 (*d*, *J* = 8.4 Hz, 2H), 3.17 (*s*, 3H), 1.52 (*m*, 6H), 1.30 (*m*, 6H), 1.06 (*t*, *J* = 7.9 Hz, 6H), 0.85 (*t*, *J* = 7.3 Hz, 9H); ^13^C-NMR (broadband proton-decoupled, 150 MHz, DMSO-*d*_6_) δ ppm: 155.48 (C), 147.04 (C), 142.60 (C), 137.77 (2CH), 136.72 (2C), 120.76 (CH), 119.39 (CH), 118.91 (2CH), 112.77 (CH), 40.75 (3CH_2_), 28.49 (CH_3_), 26.55 (3CH_3_), 13.43 (6CH_2_); FT-IR (KBr) cm^−1^; 3,431, 2,957, 2,928, 1,600; FAB-MS m/z: 599 [M + H]+; HR-FAB-MS: Calculated for C_25_H_38_N_2_O_5_SSn + H [M + H]^+^:599.1596, found 599.1607.

#### 2.2.3 *N*-{4-Nitro-2-[3-(tributylstannyl)phenoxy]phenyl}methanesulfonamide (2c).

Compound **2c** was synthesized from **1c** using the same procedure that was used to convert **1b** into **2b**. The mixture was heated at 120 °C for 3 h under reflux. The reaction mixture was allowed to cool, and a saturated aqueous solution of potassium fluoride was added. The mixture was then stirred at room temperature for 40 min. The residue was purified using chromatography on silica gel with chloroform:hexane:acetone in a 1:18:1 proportion, which yielded **2c** (13.5 mg, 48.1%) as a yellow solid: mp: 54–55 °C; ^1^H-NMR (600 MHz, DMSO-*d_6_*) δ ppm: 10.15 (*s*, 1H), 8.02 (*dd*, *J* = 2.6 Hz, 9.2 Hz, 1H), 7.72 (*d*, *J* = 9.2 Hz, 1H), 7.51 (*d*, *J* = 2.9 Hz, 1H), 7.45 (*t*, *J* = 7.7 Hz, 1H), 7.33 (*d*, *J* = 7.2 Hz, 1H), 7.18 (*s*, 1H), 7.10 (*d*, *J* = 8.1 Hz, 1H), 3.19 (*s*, 3H), 1.48 (quin, *J* = 7.7 Hz, 6H), 1.25 (sex, *J* = 7.3 Hz, 6H), 1.04 (*t*, *J* = 8.1 Hz, 6H), 0.81 (*t*, *J* = 7.3 Hz, 9H); ^13^C-NMR (broadband proton-decoupled, 150 MHz, DMSO-*d_6_*) δ ppm: 154.58 (C), 147.59 (C), 144.04 (C), 142.49 (C), 136.23 (C), 132.41 (CH), 129.73 (CH), 126.53 (CH), 120.80 (CH), 119.23 (CH), 119.05 (CH), 112.03 (CH), 40.77 (3CH_2_), 28.42 (CH_3_), 26.51 (3CH_3_), 13.53 (3CH_2_), 13.36 (3CH_2_); FT-IR (KBr) cm^−1^; 3,378, 2,958, 2,927, 2,854; FAB-MS m/z: 599 [M + H]+; HR-FAB-MS: Calculated for C_25_H_38_N_2_O_5_SSn + H [M + H]^+^:599.1596, found 599.1607.

### 2.3 Radiochemistry

Radioiodination of compounds **2b** and **2c** with iodine-125 was performed using the chloramine-T method. Tributyltin precursor **2b** or **2c** (1.4 mg, 2.34 μmol) was dissolved in 0.7 mL of 1% acetic acid in methanol. To this solution, 660 µL of a 1 mg/mL (2.34 μmol) chloramine-T solution in methanol was added, followed by an aqueous solution of [^125^I] NaI. The mixture was then stirred at room temperature for 1 min. The radioactivity of the [^125^I]NaI added at this step was individually set for each experiment within a range of 1–90 MBq. To the reaction mixture, 600 µL of purified water was added. The entire volume was applied to a semi-preparative HPLC column (COSMOSIL 5C_18_ MS-II Packed Column, 10 mm ID × 150 mm, Nacalai Tesque Inc., Kyoto, Japan, 34355-91), which was equipped with a UV absorbance detector (*λ* = 245 nm) and a radiation detector. The mobile phase consisted of a 50:50 mixture of acetonitrile (MeCN) containing 0.1% trifluoroacetic acid (TFA) and purified water containing 0.1% TFA, with a flow rate of 4 mL/min. Each [^125^I]product fraction (retention time: 16–20 min for both [^125^I]**1b** and [^125^I]**1c**) was collected and diluted fivefold with purified water. The diluted solution was passed through an activated Sep-Pak C18 Plus Light Cartridge (Waters, WAT023501) to adsorb [^125^I]**1b** or [^125^I]**1c**, which was then eluted with 500 µL of MeCN. The eluate was dried under a stream of argon gas and then dissolved in either ethanol or physiological saline containing 1% Tween 80 to prepare the solutions of [^125^I]**1b** and [^125^I]**1c** for subsequent experiments. Radiochemical purity was analyzed using HPLC: COSMOSIL 5C_18_ MS-II Packed Column 4.6 mm ID × 150 mm (Nacalai Tesque Inc., 38019-81); mobile phase, 50:50 mixture of MeCN containing 0.1% TFA and purified water containing 0.1% TFA; and flow rate of 1 mL/min. The retention times were 17.2 min for [^125^I]**1b** and 13.7 min for [^125^I]**1c**. Each labeled compound was identified as the target product by co-injection with the corresponding non-radioactive compound **1b** or **1c**, which confirmed matching retention times.

### 2.4 Biodistribution of radioactivity following the administration of radiotracers in mice

Each radiotracer was intravenously injected. The ^125^I radioactivity in the samples obtained was counted with the auto-well gamma counter, and the tissues were weighed. The tissue uptake of ^125^I was expressed as the percentage of the injected dose per gram of tissue (%ID/g), except for the thyroid, for which it was expressed as the percentage of the injected dose per organ.

#### 2.4.1 Tissue distribution in normal mice

Male BALB/cCrSlc mice (8 weeks old, body weight 20–24 g) were euthanized by cervical dislocation 30 min and 1, 2, 6, 12, and 24 h after the radiotracer (800 kBq/0.2 mL/head) injection. Sample sizes of [^125^I]**1b** were *n* = 4 for 30 min, *n* = 5 for 1 h and 6 h, *n* = 6 for 12 h and 24 h, and *n* = 8 for 2 h. Sample sizes of [^125^I]**1c** were *n* = 5 for 12 h; *n* = 6 for 2 h, 6 h, and 24 h; *n* = 7 for 30 min; and *n* = 9 for 1 h.

#### 2.4.2 Tissue distribution in a mouse model of inflammation

A mouse model of inflammation was established using a previously described procedure with slight modifications (22). For developing the inflammation model, male BALB/cCrSlc mice (7 weeks old) were anesthetized with a 1.5% isoflurane air mixture. A paper disk (for antibiotic, *ϕ* = 8 mm, #49005020, Advantec Toyo Kaisha, Ltd., Tokyo, Japan) soaked in turpentine was subcutaneously inserted into the thigh of the right hind leg. Seven days after the turpentine treatment, [^125^I]**1b** or [^125^I]**1c** (600–900 kBq/0.2 mL/head) was intravenously injected into the mouse model of inflammation (8 weeks old, body weight 15–22 g). The mice were euthanized by cervical dislocation 30 min and 1, 2, 6, 12, and 24 h after radiotracer injection, and the radioactivity levels were evaluated. Sample sizes of [^125^I]**1b** were *n* = 6 for 1 h, 2 h, and 24 h; *n* = 7 for 6 h and 12 h; and *n* = 10 for 30 min. Sample sizes of [^125^I]**1c** were *n* = 6 for 30 min, 2 h, 6 h, and 24 h, and *n* = 7 for 1 h and 12 h.

#### 2.4.3 Blocking study in a mouse model of inflammation

The mouse model of inflammation (8 weeks old, body weight 18–23 g) received blocker solutions (1 mg/kg body weight, 0.1 mL) of nimesulide, non-radioactive **1b**, indomethacin, and celecoxib dissolved in DMSO, which were co-injected with [^125^I]**1b** (800–900 kBq/0.1 mL/head). The control mice received the same volume of DMSO. The mice were euthanized by cervical dislocation 6 h after the radiotracer injection, and the radioactivity levels were measured. Sample sizes were *n* = 4 for nimesulide and celecoxib, *n* = 5 for indomethacin, and *n* = 6 for the **1b** and the control groups.

### 2.5 *Ex vivo* autoradiography in mice

[^125^I]**1b** or [^125^I]**1c** (6.1–7.5 MBq, *n* = 1) were intravenously injected into BALB/cCrSlc mice (8 weeks old, body weight 22–25 g). The mice were euthanized by cervical dislocation at 30 min and 1, 2, 3, 6, 12, and 24 h after radiotracer injection. The brain was rapidly dissected, frozen, and embedded in Tissue-Tek O.C.T. Compound (Sakura Finetek Japan Co., Ltd., Tokyo, Japan, 45833) before being sectioned coronally at a thickness of 10 *μ*m using a CryoStar NX50 (Epredia holdings Ltd., Kalamazoo, MI, USA). Coronal sections were prepared relative to the mouse brain bregma at 1.0 mm anterior, at the bregma level, 1.4 mm posterior, and 2.1 mm posterior. The sections were dried and exposed to Storage Phosphor Screen BAS-IP MS 2025 (Cytiva, Marlborough, MA, USA, 28956475). After 3 weeks of autoradiographic exposure, the distribution of ^125^I was visualized using a fluorescent image analyzer (FLA3000; formerly FUJIFILM, Cytiva).

Autoradiographic imaging was conducted at 6 h after administration of [^125^I]**1b**, with or without inhibitor treatment, using the same procedure. BALB/cCrSlc mice (10 weeks old, body weight 26 g, *n* = 1) were co-administered [^125^I]**1b** (6.3–6.4 MBq/head) together with indomethacin or celecoxib dissolved in DMSO (1 mg/kg body weight, 0.05 mL). In control mice, the same volume of DMSO was co-injected. After 2 weeks of autoradiographic exposure, the distribution of ^125^I was examined using a fluorescent image analyzer.

### 2.6 Immunohistochemistry and hematoxylin–eosin staining

Male BALB/cCrSlc mice (8 weeks old, body weight 24–25 g, *n* = 2) were anesthetized with pentobarbital (7.1 mg/mL) administered intraperitoneally at 0.01 mL per gram body weight. Under deep anesthesia, the animals were perfused with 15 mL of PBS, followed by fixation with 35 mL of 4% paraformaldehyde in PBS. The whole brain was removed and stored in 4% paraformaldehyde in PBS at 4 °C. Subsequently, coronal sections were cut at 1, 2, 3.5, 4.5, and 5.5 mm from the frontal pole, embedded, and processed into paraffin blocks using PathoprepR546 and Pathoprep568 (FUJIFILM Wako Pure Chemical Corp., Osaka, Japan). Paraffin blocks were sectioned at a thickness of 8 μm from the tissue surface using a microtome. The sections were mounted onto glass slides, stretched for approximately 1 min on a 50 °C warming plate, and then dried overnight at 45°C. The locations of the sections were converted to distances from bregma to correspond to the positions of the autoradiographic sections.

For hematoxylin and eosin staining, the prepared sections were deparaffinized, rinsed with water, and stained with hematoxylin solution. They were then washed in running water for 10 min and stained with eosin solution. After a brief rinse in water, the sections were dehydrated sequentially with 70% and 90% ethanol, followed by three changes of 100% ethanol. Finally, the sections were cleared in xylene (three times) and mounted.

For immunohistochemical staining, the prepared sections were deparaffinized in xylene, rehydrated through graded alcohols, and rinsed three times with PBS. The sections were then immersed in citrate buffer and subjected to antigen retrieval by heating at 121 °C for 5 min in an autoclave. The sections were rinsed three times with PBS, then treated with 3% hydrogen peroxide in methanol for 10–15 min at room temperature to block endogenous peroxidase activity, followed by three additional washes with PBS. The COX-2 antibody (COX-2 (mouse) Polyclonal Antibody (aa 570–598), 160106, lot:0617220-1, Cayman Chemical, Ann Arbor, MI, USA) was diluted 1:500 in antibody dilution buffer and incubated with the sections overnight at 4°C. The sections were rinsed three times with PBS, incubated with the primary antibody (HISTFINE Simple Stain Mouse MAX-PO(R), 414341, NICHIREI BIOSCIENCES Inc., Tokyo, Japan) for 30 min at room temperature, and then washed three times with PBS. Staining was developed using 3,3′-diaminobenzidine tetrahydrochloride for 5 min. The sections were rinsed under running water, counterstained with hematoxylin for 30 s, washed again, dehydrated, cleared, and mounted.

### 2.7 Metabolite analysis

Each of the radiotracers (6–8 MBq for [^125^I]**1b**, 10–20 MBq for [^125^I]**1c**, *n* = 3–4) was intravenously injected into BALB/cCrSlc mice or the mouse model of inflammation (8–9 weeks old, body weight 18–24 g). Mice were euthanized by cervical dislocation at 6, 12, and 24 h after radiotracer injection. For normal mice, additional euthanizations were performed at 15 and 30 min post-injection. Blood was collected by cardiac puncture using a heparinized syringe at each time point. In normal mice, the brain was removed, whereas in the mouse model of inflammation, the inflamed region was removed, and all tissues were kept on ice until further processing. The blood was centrifuged at 2,000 × *g* for 1 min at 4°C to obtain the plasma, which (50 μL) was denatured with 200 μL of 50 mM AcOH–AcONH_4_ aqueous buffer (50:50) and 250 μL of MeCN. The mixture was centrifuged under the same conditions, and the precipitate was resuspended in 500 μL of a 1:1 mixture of MeCN and 50 mM AcOH–AcONH_4_ aqueous buffer (50:50), followed by centrifugation. This procedure was repeated twice. The whole brain was homogenized in 1,000 μL of a 1:1 mixture of MeCN and 50 mM AcOH–AcONH_4_ aqueous buffer (50:50). The homogenate was then processed using the same procedure as was followed for processing the plasma sample. The inflamed region was homogenized in 1,000 μL of 50 mM AcOH–AcONH_4_ aqueous buffer. To 250 μL of the homogenate, 250 μL of MeCN was added, and the resulting mixture was processed to prepare analytical samples in the same manner as the plasma samples. After protein elimination, the recovery of the radioactivity in the soluble fractions was 67%–99%. The combined supernatant was analyzed using HPLC: COSMOSIL 5C_18_ MS-II Packed Column 10 mm ID × 250 mm (Nacalai Tesque Inc., 38023-11). The mobile phase comprised a mixture of MeCN:50 mM aqueous AcOH:50 mM aqueous AcONH_4_ in a 51:24.5:24.5 (v/v/v) ratio at a flow rate of 4 mL/min. Fractions were collected at 30-s intervals, and their radioactivity was measured using the auto-well gamma counter.

### 2.8 Protein binding

The protein binding of [^125^I]**1b** was evaluated with modifications based on a previously reported method (26–28), and the conditions were optimized experimentally. [^14^C]Diazepam, [^14^C]antipyrine, and [^3^H]cyclosporin A were purchased from American Radiolabeled Chemicals, Inc. (Saint Louis, MO, USA, ARC1442, ARC0108, ART1717). [^14^C]Diazepam and [^14^C]antipyrine were diluted with D-PBS (–) to prepare solutions at a final concentration of 250 nM. [^3^H]Cyclosporin A was prepared by adding non-radioactive Cyclosporin A and diluting with HBSS to achieve a final concentration of 250 nM. The ethanol solution of [^125^I]**1b** was diluted with D-PBS (–) to prepare a solution with a radioactivity of 1 MBq/mL. Human plasma (COSMO BIO Co., Ltd., Tokyo, Japan, KOJ-12250210, lot: BJ18665A) and mouse plasma (Rockland Immunochemicals, Inc., Limerick, PA, USA, D408-06-0050, lot:50597) were filtered using syringe filters with polyether sulfone membranes (Millex-HP, 0.45 μm, PES 33 mm, MilliporeSigma, Burlington, MA, USA, SLHP033NS) to remove fibrinogen. Human albumin (Sigma-Aldrich Inc., A3782, lot: 1003579845) and human *α*1-acid glycoprotein (Sigma-Aldrich Inc., G9885, lot: 1003617600) were dissolved in D-PBS (–) to prepare solutions at concentrations of 700 μM and 30 μM, respectively. Protein binding assays were performed in low-binding microtubes (ProteoSave microtube 0.5 mL, Sumitomo Bakelite Co., Ltd., Tokyo, Japan, MS-4205M) to minimize protein adsorption to the container. Ultrafiltration was performed using either Centrifree ultrafiltration devices (30 kDa, MilliporeSigma, 4104) or Amicon Ultra-0.5 centrifugal filter units (30 kDa, MilliporeSigma, UFC503096). Centrifugation was performed at 1,923 *×* *g* for Centrifree devices and at 14,211 × *g* for Amicon filters, both at 20°C for 40 min. The radioactivity of ^125^I was measured using an auto-well gamma counter, while the radioactivity of ^3^H and ^14^C was measured with a liquid scintillation counter (AccuFLEX LSC-8000, Aloka) after mixing the samples with 3 mL of Clear-Sol I (Nacalai Tesque Inc., 09135-93).

To 250 μL of prepared human plasma, mouse plasma, human albumin solution, or human *α*1-acid glycoprotein solution, 25 μL of the radiolabeled solution [[^14^C]diazepam, [^14^C]antipyrine, or [^125^I]**1b**] was added (*n* = 6). The reaction mixture was then incubated in a water bath at 37°C for 15 min. After incubation, half of the reaction mixtures (*n* = 3 out of 6) were transferred to either Centrifree ultrafiltration devices or Amicon Ultra centrifugal filters for ultrafiltration. The radioactivity of the reaction mixtures that were not subjected to ultrafiltration (*n* = 3) and of the filtrates after ultrafiltration (*n* = 3) was measured to determine the fraction of unbound drugs, from which the percentage of protein binding was calculated. For Amicon filters, the protein binding of [^3^H]cyclosporin A was also evaluated using the same procedure.

### 2.9 Statistical analysis

Statistical analyses were performed using Excel 2021 (Microsoft, Redmond, WA, USA). Quantitative data are expressed as the mean ± standard deviation (SD), except for datasets with *n* = 2, which are presented as mean values only. A *p*-value of <0.05 was considered statistically significant.

In the blocking study using a mouse model of inflammation, one-way analysis of variance followed by Dunnett's test was conducted for each organ. Dunnett's test was applied to compare each inhibitor group against the control group. For the inflammation/blood and inflammation/muscle ratios, data were subjected to natural logarithmic transformation prior to analysis to stabilize variances; the statistical tests were therefore conducted using the log-transformed ratios.

## 3 Results

### 3.1 Chemistry

**Scheme 1:**
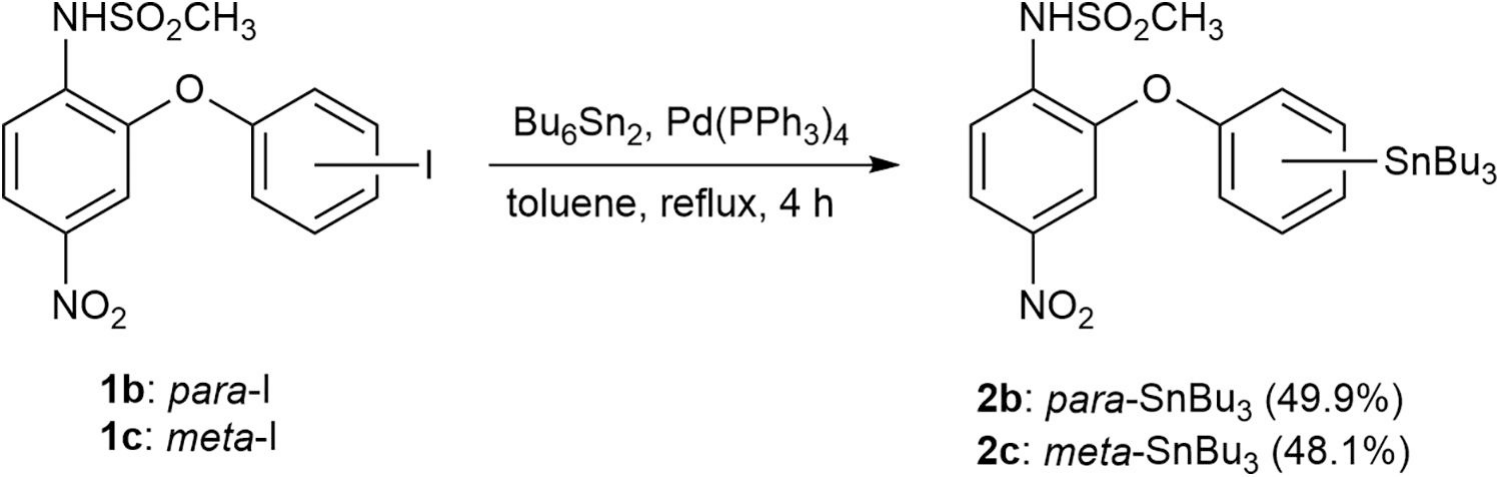
Synthetic pathway for tributyltin precursors. The numbers in parentheses following each compound indicate the reaction yield (%).

Nimesulide (**1a**) and its isomeric iodinated derivatives **1b** and **1c** were prepared as previously described (24). Tributylstannyl derivatives **2b** and **2c,** required for ^125^I-radiolabeling, were prepared via stannylation of the corresponding iodinated compounds **1b** and **1c** (Scheme 1). The structures and structural integrity of all compounds were rigorously confirmed by ^1^H-NMR and high-resolution mass spectrometry.

### 3.2 Radiochemistry

**Scheme 2:**
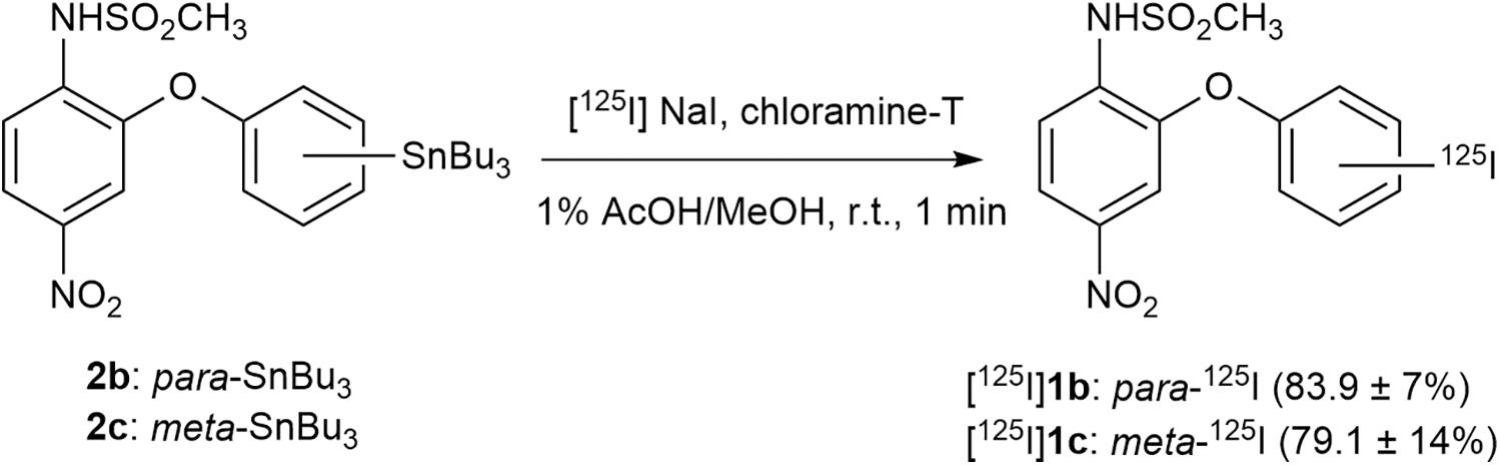
Radiosynthesis of ^125^I-labeled nimesulide iodinated analogs. The numbers in parentheses following each compound represent the radiochemical yield as mean ± SD (%). For [^125^I]**1b**, *n* = 8, and for [^125^I]**1c**, *n* = 6.

Radiosynthesis of [^125^I]**1b** and [^125^I]**1c** was accomplished as shown in Scheme 2. [^125^I]**1b** and [^125^I]**1c** were synthesized by radioiodination of the tributylstannyl derivatives **2b** and **2c** with [^125^I]NaI in the presence of chloramine-T in 1% acetic acid in methanol, stirred at room temperature for 1 min. The oxidizing agents N-chlorosuccinimide and chloramine-T were compared for this reaction. Although both agents enabled successful radiolabeling, the radiochemical yields for [^125^I]**1b** were approximately 70% with N-chlorosuccinimide and approximately 85% with chloramine-T, whereas those for [^125^I]**1c** were approximately 45% and 65%, respectively, clearly indicating that chloramine-T is the more suitable oxidizing agent for both compounds. The reaction progress was monitored up to 120 min after initiation. Notably, more than 80% of [^125^I]**1b** and more than 60% of [^125^I]**1c** were formed within 15 s. Considering ease of handling, a final reaction time of 1 min was adopted for all subsequent experiments. After establishing the radiolabeling conditions, the radiolabeling of [^125^I]**1b** was performed eight times, and that of [^125^I]**1c** six times in the experiments reported here. The radiochemical yields were 83.9 ± 7% and 79.1 ± 14%, respectively, and the radiochemical purities were consistently greater than 99% in both cases.

### 3.3 Biodistribution of radioactivity following the administration of radiotracers in normal mice

To assess *in vivo* behavior, the tissue distribution of the radioactivity after injecting [^125^I]**1b** and [^125^I]**1c** into normal male BALB/cCrSlc mice was determined and is summarized in Tables 1A, 1B. The full version of the data is provided in the Supplementary Materials (Supplementary Tables S1A, S1B). In the whole brain, the radioactivity of [^125^I]**1b** reached a maximum of 1.16 %ID/g at 2 h after administration, while that of [^125^I]**1c** peaked at 1.38 %ID/g at 1 h. However, notably, in both cases, these brain uptake levels were lower than those observed in blood and muscle. The radioactivity of [^125^I]**1b** remained in the bloodstream for an extended period, maintaining remarkably high blood retention even 24 h after administration, whereas the radioactivity of [^125^I]**1c** showed high blood accumulation at 30 min post-injection followed by time-dependent clearance. However, the radioactivity of [^125^I]**1c** was also cleared over time from muscle and brain, whereas its thyroid accumulation increased substantially from 0.10 %ID/g at 2 h to 0.49 %ID/g at 24 h after administration, suggesting *in vivo* deiodination. Thyroid accumulation of the radioactivity of [^125^I]**1b** was 0.09 %ID/g at 30 min and 0.10 %ID/g at 24 h after administration, showing no substantial change over time.

**Table 1A:** Biodistribution study of [^125^I]**1b** in BALB/c mice^a^.

Tissue	Uptake (%ID/g)^b^					
	30 min	1 h	2 h	6 h	12 h	24 h
Blood	13.99 ± 3.7	12.39 ± 1.9	11.48 ± 2.2	12.62 ± 2.1	10.72 ± 1.7	7.82 ± 1.2
Muscle	2.56 ± 0.8	5.63 ± 4.3	4.71 ± 3.2	2.97 ± 1.7	2.17 ± 0.8	1.53 ± 0.4
Brain	0.89 ± 0.3	1.00 ± 0.2	1.16 ± 0.2	1.14 ± 0.3	1.00 ± 0.1	0.47 ± 0.1
Thyroid^c^	0.09 ± 0.0	0.08 ± 0.0	0.06 ± 0.0	0.08 ± 0.0	0.09 ± 0.0	0.10 ± 0.0

a The full version of the data is provided in the Supplementary Materials.

b Data are presented as percent injected dose per gram of tissue (mean ± standard deviation). Sample sizes were *n* = 4 for 30 min; *n* = 5 for 1 h and 6 h; *n* = 6 for 12 h and 24 h; and *n* = 8 for 2 h.

c Data are presented as percent injected dose per organ.

**Table 1B:** Biodistribution study of [^125^I]**1c** in BALB/c mice^a^.

Tissue	Uptake (%ID/g)^b^					
	30 min	1 h	2 h	6 h	12 h	24 h
Blood	10.59 ± 2.0	8.58 ± 1.0	6.99 ± 1.5	3.09 ± 0.6	0.62 ± 0.1	0.14 ± 0.0
Muscle	7.72 ± 4.1	2.33 ± 0.7	1.59 ± 0.3	1.66 ± 1.2	0.16 ± 0.0	0.04 ± 0.0
Brain	1.30 ± 0.1	1.38 ± 0.3	1.27 ± 0.2	0.51 ± 0.1	0.11 ± 0.0	0.01 ± 0.0
Thyroid^c^	0.05 ± 0.0	0.05 ± 0.0	0.10 ± 0.0	0.43 ± 0.1	0.42 ± 0.2	0.49 ± 0.1

a The full version of the data is provided in the Supplementary Materials.

b Data are presented as percent injected dose per gram of tissue (mean ± standard deviation). Sample sizes were *n* = 5 for 12 h; *n* = 6 for 2 h, 6 h, and 24 h; *n* = 7 for 30 min; and *n* = 9 for 1 h.

c Data are presented as percent injected dose per organ.

### 3.4 *Ex vivo* autoradiography in mice and immunohistochemistry and hematoxylin–eosin staining

**Figure 2:**
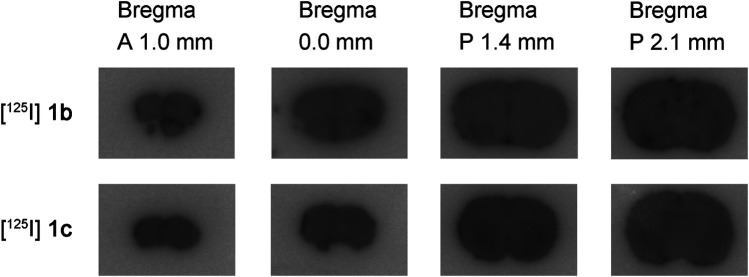
Representative *ex vivo* autoradiographic brain images (coronal) in BALB/c mice at 2 h after administration of compounds [^125^I]**1b** and [^125^I]**1c.** Coronal sections were prepared relative to the mouse brain bregma at 1.0 mm anterior (A 1.0 mm), at the bregma level (0.0 mm), 1.4 mm posterior (P 1.4 mm), and 2.1 mm posterior (P 2.1 mm).

**Figure 3:**
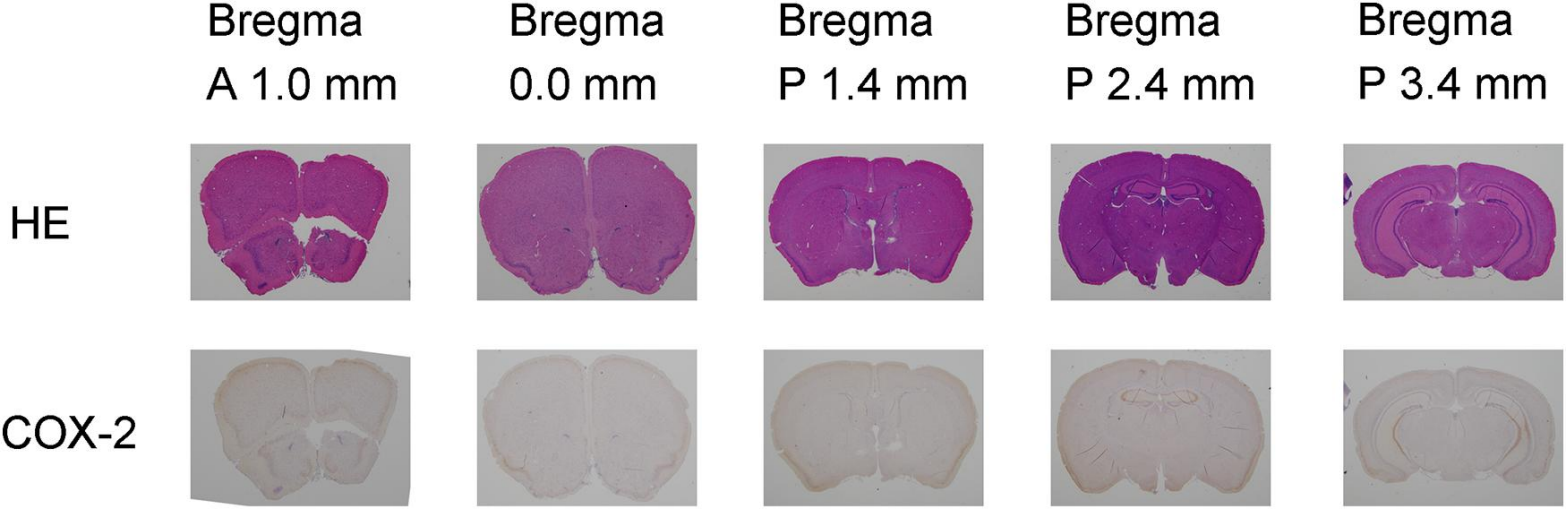
Representative coronal brain sections of BALB/c mice stained with hematoxylin–eosin (HE) and using immunohistochemistry with a COX-2 antibody (COX-2). Coronal sections were prepared relative to the mouse brain bregma at 1.0 mm anterior (A 1.0 mm), at the bregma level (0.0 mm), 1.4 mm posterior (P 1.4 mm), 2.4 mm posterior (P 2.4 mm), and 3.4 mm posterior (P 3.4 mm).

To visualize the regional brain distribution, representative *ex vivo* autoradiographic images of mouse brain coronal sections taken 2 h after administration are shown in Figure 2. For comparison with mouse brain autoradiography, hematoxylin–eosin staining and immunohistochemical staining with a COX-2 antibody of coronal sections are presented in Figure 3. The radioactivity of [^125^I]**1b** was uniformly distributed across all section levels, with areas of higher intensity appearing to correspond to regions containing blood, rather than specific neural structures. At 6 h after co-administration of [^125^I]**1b** with the inhibitors (indomethacin or celecoxib), the distribution of radioactivity did not show any appreciable differences from that in the control sections at any coronal level, indicating a lack of specific, blockable binding (data not shown). The radioactivity distribution of [^125^I]**1c** was generally similar to that of [^125^I]**1b**, with a uniform pattern across sections; however, slight regional variations in accumulation were observed in the section located 1.4 mm posterior to bregma (Bregma P 1.4 mm). An initial comparison of the regions showing slight accumulation of the radioactivity of [^125^I]**1c** with hematoxylin–eosin-stained sections suggests that the areas of accumulation correspond to the striatum. Similar results were observed at other time points (from 15 min to 12 h post-injection), which are not shown in the figures. Immunohistochemical staining with a COX-2 antibody revealed that COX-2 is expressed in the marginal regions of the cerebral cortex and in the hippocampus of BALB/cCrSlc mouse brain.

### 3.5 Biodistribution of radioactivity following the administration of radiotracers in a mouse model of inflammation

To evaluate the potential for imaging inflammation, the distribution of radioactivity of [^125^I]**1b** in the mouse model of inflammation is summarized in Table 2. The data for [^125^I]**1c** and the full dataset for [^125^I]**1b** are provided in the Supplementary Materials (Supplementary Tables S2A, S2B). The uptake of radioactivity of [^125^I]**1b** in the inflammatory region increased over time, peaked at 6 h post-administration (7.54 %ID/g), and subsequently decreased gradually until 24 h. At 6 h post-administration, when the accumulation in the inflammatory region reached its peak, the distribution of radioactivity of [^125^I]**1b** with or without various COX inhibitors is shown in Tables 3A, 3B. Notably, the uptake of radioactivity of [^125^I]**1b** in the inflammatory region and the inflammation-to-blood ratio were not significantly reduced by any of the COX inhibitors compared with the control, again suggesting the signal was not specific to COX-2. In contrast, the inflammation-to-muscle ratio of radioactivity of [^125^I]**1b** was significantly reduced by nimesulide and celecoxib compared with the control, indicating a more complex interaction in peripheral tissues.

**Table 2:** Biodistribution study of [^125^I]**1b** in a mouse model of inflammation^a^.

Tissue	Uptake (%ID/g)^b^					
	30 min	1 h	2 h	6 h	12 h	24 h
Blood	11.74 ± 2.8	14.52 ± 1.6	14.07 ± 2.7	12.65 ± 1.9	9.33 ± 1.6	9.00 ± 1.2
Muscle	2.75 ± 1.5	2.31 ± 0.6	2.53 ± 0.2	1.93 ± 0.4	1.44 ± 0.4	1.37 ± 0.3
Inflammation	4.97 ± 1.4	5.79 ± 1.7	7.20 ± 1.9	7.54 ± 1.6	4.29 ± 0.7	4.81 ± 1.0
Thyroid^c^	0.08 ± 0.0	0.08 ± 0.0	0.08 ± 0.0	0.10 ± 0.0	0.09 ± 0.1	0.14 ± 0.0

a The full version of the data is provided in the Supplementary Materials.

b Data are presented as percent injected dose per gram of tissue (mean ± standard deviation). Sample sizes were *n* = 6 for 1 h, 2 h, and 24 h; *n* = 7 for 6 h and 12 h; and *n* = 10 for 30 min.

c Data are presented as percent injected dose per organ.

**Table 3A:** Biodistribution study of [^125^I]**1b** with or without cyclooxygenase inhibitors 6 h after injection in a mouse model of inflammation^a^.

Tissue	Uptake (%ID/g)^b^				
	Control	Nimesulide	**1b**	Indomethacin	Celecoxib
Blood	12.96 ± 2.3	12.06 ± 0.5	13.14 ± 3.2	16.48 ± 0.6	14.39 ± 1.5
Muscle	2.04 ± 0.5	2.88 ± 0.4	2.88 ± 0.7	2.78 ± 0.8	3.82 ± 0.9
Inflammation	6.25 ± 1.0	5.82 ± 1.3	7.09 ± 2.1	6.52 ± 0.8	6.45 ± 1.1
Thyroid^c^	0.10 ± 0.0	0.09 ± 0.0	0.10 ± 0.0	0.12 ± 0.1	0.07 ± 0.0

a The full version of the data is provided in the Supplementary Materials.

b Data are presented as percent injected dose per gram of tissue (mean ± standard deviation). Sample sizes were *n* = 4 for nimesulide and celecoxib, *n* = 5 for indomethacin, and *n* = 6 for **1b** and the control group.

c Data are presented as percent injected dose per organ.

**Table 3B:** Inflammation to tissue ratio of [^125^I]**1b** with or without cyclooxygenase inhibitors 6 h after injection in a mouse model of inflammation.

Ratio type	Ratio^a^				
	Control	Nimesulide	**1b**	Indomethacin	Celecoxib
Inflammation/blood	0.49 ± 0.1	0.48 ± 0.1	0.54 ± 0.1	0.40 ± 0.1	0.45 ± 0.1
Inflammation/muscle	3.12 ± 0.5	2.05 ± 0.6*	2.46 ± 0.3	2.47 ± 0.5	1.76 ± 0.5*

a Data are presented as mean ± standard deviation. Sample sizes were *n* = 4 for nimesulide and celecoxib, *n* = 5 for indomethacin, and *n* = 6 for **1b** and the control group.

* Significant decrease (*p* < 0.05) compared to the control (Dunnett's test).

### 3.6 Metabolite analysis

To investigate the metabolic stability *in vivo*, the time-dependent changes in the percentage of unchanged compounds [^125^I]**1b** and [^125^I]**1c** after administration to BALB/cCrSlc mice are shown in Table 4A. Similarly, the time-dependent changes in the percentage of unchanged compounds [^125^I]**1b** and [^125^I]**1c** after administration to the mouse model of inflammation are shown in Table 4B. In both normal mice and the mouse model of inflammation, the percentage of unchanged [^125^I]**1b** in plasma remained remarkably stable, staying above 85% up to 24 h post-administration. At 24 h, the percentage of unchanged [^125^I]**1b** in the brain was 82%, while that in the inflammatory region was 78%. In sharp contrast, the percentage of unchanged [^125^I]**1c** in plasma decreased considerably to 42% at 24 h post-administration in normal mice and to 29% in the mouse model of inflammation. Furthermore, at 24 h post-administration, the percentage of unchanged [^125^I]**1c** was only 24% in the brain of normal mice and 41% in the inflammatory region of the mouse model of inflammation.

**Table 4A:** Metabolite analysis of compounds [^125^I]**1b** and [^125^I]**1c** in BALB/c mice: percentage of unchanged forms at multiple time points after administration.

Time after injection	Percentage of unchanged forms (%)^a^			
	[^125^I] **1b**		[^125^I] **1c**	
	Plasma	Brain	Plasma	Brain
15 min	97 ± 0	77 ± 4	90 ± 4^b^	43 ± 14^b^
30 min	93 ± 5	63 ± 18	86 ± 5	65 ± 21
6 h	85 ± 14	83 ± 7	83 ± 3	69 ± 7
12 h	91 ± 1	84 ± 1	76 ± 3	59 ± 3
24 h	89 ± 2	82 ± 3	42 ± 8	24 ± 5

a Data are presented as mean ± standard deviation. Sample sizes were *n* = 3.

b Sample size was *n* = 4.

**Table 4B:** Metabolite analysis of compounds [^125^I]**1b** and [^125^I]**1c** in a mouse model of inflammation: percentage of unchanged forms at multiple time points after administration.

Time after injection	Percentage of unchanged forms (%)^a^			
	[^125^I] **1b**		[^125^I] **1c**	
	Plasma	Inflammation	Plasma	Inflammation
6 h	92^b^	72 ± 13	72 ± 15	74 ± 9
12 h	88^b^	74 ± 6	65 ± 10	70 ± 10
24 h	86 ± 3	78 ± 3	29 ± 6	41 ± 6

a Data are presented as mean ± standard deviation. Sample sizes were *n* = 3.

b Data are presented as the mean of two determinations. Sample size was *n* = 2.

### 3.7 Protein binding

Given the high blood retention observed *in vivo*, plasma protein binding for [^14^C]diazepam, [^14^C]antipyrine, and [^125^I]**1b** was determined *in vitro* using Centrifree devices, with the results presented in Table 5. In addition to these compounds, the percentages of protein binding determined using Amicon filters, including [^3^H]cyclosporin A, are shown in the Supplementary Materials (Supplementary Table S4). As a positive control, [^14^C]diazepam, a drug with high protein binding, showed protein binding percentages of 99.0% in human plasma and human albumin, 93.6% in mouse plasma, and 50.2% in human *α*1-acid glycoprotein. As a negative control, [^14^C]antipyrine, a drug with low protein binding, exhibited protein binding percentages of 32%–44% in human plasma, mouse plasma, and human albumin, and 1.5% in human *α*1-acid glycoprotein. In comparison, [^125^I]**1b** exhibited exceptionally high protein binding, with percentages of 99.9% in human plasma, mouse plasma, and human albumin, and 76.5% in human *α*1-acid glycoprotein. These results were found to be consistent when assessed using Amicon filters, which offer a simpler operation compared with Centrifree devices, thereby validating the finding across different methodologies.

**Table 5:** Determination of protein binding percentages for [^14^C]diazepam, [^14^C]antipyrine, and [^125^I]**1b**.

Sample type	Percentage of protein binding (%)		
	[^14^C]Diazepam	[^14^C]Antipyrine	[^125^I]**1b**
Human plasma	99.0 ± 0.2	44.3 ± 2.0	99.9 ± 0.0
Mouse plasma	93.6 ± 0.3	37.0 ± 1.0	99.9 ± 0.0
Human albumin	99.0 ± 0.3	32.6 ± 3.0	99.9 ± 0.0
Human *α*1-acid glycoprotein	50.2 ± 4.2	1.5 ± 12.7	76.5 ± 1.3

Protein binding percentages were determined using Centrifree ultrafiltration devices (*n* = 3). Data obtained with Amicon devices are provided in the Supplementary Materials.

## 4 Discussion

The primary objective of this study was to address the critical need for brain-penetrant COX-2 imaging agents by evaluating novel radioiodinated nimesulide analogs, initially using iodine-125 for assessment. However, the results showed that the developed tracers did not achieve the requisite brain uptake for this initial goal, and the brain radioactivity distribution did not correspond to the COX-2 distribution determined in this study, suggesting limited potential as brain-targeted imaging agents at this stage. Nevertheless, this investigation led to a significant and serendipitous discovery: of the *para*-iodo derivative, [^125^I]**1b**, which possesses COX-2 inhibitory activity, demonstrated considerable promise as a highly stable, specific agent for imaging COX-2 in peripheral inflammation, and this specificity was further supported by the observation that its accumulation could be blocked by a known COX-2 inhibitor. These results do not preclude the potential of this compound as a brain-targeted imaging agent; however, this pivot from a CNS to a peripheral application represents a key and unexpected outcome of our work, opening a new and valuable avenue for this class of compounds.

In this study, we successfully synthesized the tributyltin derivatives **2b** and **2c**, corresponding to compound **1b**, which possesses COX-2 inhibitory activity, and its isomer **1c**, which lacks such activity, respectively, as precursors for ^125^I labeling, achieving satisfactory yields. All synthesized compounds yielded satisfactory spectroscopic data, fully consistent with the structures as depicted. ^125^I radiolabeling was successfully achieved, yielding both satisfactory radiochemical yields and high radiochemical purity. Strategically, [^125^I]**1b** was evaluated as a COX-2-specific SPECT imaging agent, while [^125^I]**1c**, lacking COX-2 inhibitory activity, was used as a negative control to assess non-specific accumulation and metabolic behavior.

The biodistribution data (Table 1A) suggest that the brain penetration of [^125^I]**1b** was somewhat limited, likely due to its strong binding to blood proteins, as indicated by the protein binding evaluation. Nevertheless, [^125^I]**1b** showed improved brain uptake compared with a previously reported ^11^C-labeled *para*-methoxy-nimesulide derivative in BALB/c mice, which reached a maximum brain uptake of 0.918 %ID/g (SUV; standardized uptake value 0.34) at 1 min post-injection (22), representing a modest but encouraging step forward. The biodistribution data for [^125^I]**1c** (Table 1B) showed that while radioactivity in mouse tissues decreased over time, accumulation in the thyroid increased, suggesting that [^125^I]**1c** undergoes deiodination *in vivo*. This hypothesis is further supported by the results of the metabolite analysis. In contrast, [^125^I]**1b** maintained low accumulation in the thyroid, and the results of the metabolite analysis indicate that it is remarkably stable *in vivo*.

*Ex vivo* autoradiography (Figure 2) demonstrated that the distribution of [^125^I]**1b** in the brain was uniform, and this pattern was not altered by COX inhibitors, indicating that COX-2–specific accumulation in the brain was negligible. These findings further suggest the difficulty of utilizing [^125^I]**1b** as a brain-targeted imaging agent. However, immunohistochemical staining (Figure 3) indicated that COX-2 expression in the brains of BALB/cCrSlc mice was relatively low, suggesting that the expression level may have been insufficient to adequately assess the suitability of [^125^I]**1b** as a COX-2 imaging agent. In fact, a study published in 2025 compared the uptake of [^11^C]MC1 between humanized COX-2–expressing (hCOX-2) mice and wild-type mice and reported that the wild-type mice exhibited minimal uptake (9). Therefore, further evaluation in models with higher COX-2 expression may be required to conclusively determine its potential for brain imaging. The isomer [^125^I]**1c**, which lacks COX-2 inhibitory activity, tended to accumulate in the striatum. The striatum is a region where the BBB is relatively intact under normal conditions, in the absence of pathology (29, 30). Since normal mice were used in this study, the observed accumulation of radioactivity of [^125^I]**1c** may have resulted from multiple factors, including non-specific background, local metabolism, transport mechanisms, or transient retention due to differences in blood flow. Autoradiography showed striatal accumulation from 15 min to 12 h post-injection. Concurrently, metabolite analysis over the same period indicated that 43%–69% of [^125^I]**1c** in the brain remained unchanged, suggesting that metabolites may have contributed to the observed accumulation.

The biodistribution study of [^125^I]**1b** in a mouse model of inflammation (Tables 2, 3A, 3B) demonstrated that [^125^I]**1b** selectively accumulated at the site of inflammation, and this accumulation was significantly reduced by celecoxib and nimesulide, as indicated by the inhibition of the inflammation-to-muscle ratio. Turpentine-induced inflammation elicits COX-2 expression in activated neutrophils and macrophages and is therefore commonly employed for the evaluation of COX-2–targeted radiotracers, supporting the reliability of the present findings (31–33). In this context, in the same inflammation model, the inflammation-to-muscle ratio for [^67^Ga]citrate, a commonly used inflammation imaging agent, was 4.16 ± 0.5 at 24 h post-injection, whereas that for the [^11^C]*para*-methoxy-nimesulide derivative exceeded 1.0 at all evaluated time points but remained below 2.5 (22). In comparison, [^125^I]**1b** exhibited an inflammation-to-muscle ratio above 2.1 at all time points, reaching a maximum of 4.0 at 6 h post-injection, demonstrating improved accumulation relative to the previously evaluated [^11^C]*para*-methoxy-nimesulide derivative and approaching the levels observed with the inflammation imaging agent [^67^Ga]citrate. These results indicate that [^125^I]**1b** provides sufficient contrast for COX-2–targeted SPECT imaging in the periphery. However, its prolonged retention in the blood may limit its applicability for evaluating inflammation in highly perfused organs, such as the liver.

The metabolite analysis presented in Tables 4A, 4B showed that, in both normal and inflammation model mice, [^125^I]**1b** remained largely unchanged 24 h after administration, indicating high metabolic stability, whereas [^125^I]**1c** was progressively metabolized over time. In addition, *in vivo* biodistribution data revealed that the I-125 released from [^125^I]**1c** accumulated in the thyroid. These findings demonstrate that [^125^I]**1b** is stable as an imaging agent, and the difference in metabolic stability between [^125^I]**1b** and [^125^I]**1c** provides important information for future radiotracer design. When compared with previously evaluated isomeric [^11^C]methoxy-nimesulide derivatives, the *ortho*-methoxy-nimesulide derivative showed relatively high stability, whereas the *meta*- and *para*-derivatives were rapidly metabolized. Notably, the *para*-derivative was less stable than the *meta*-derivative (22). Previously, it was shown that the major metabolite of the parent nimesulide skeleton was *para*-hydroxy nimesulide (34, 35). In our previous work, it was proposed that the 4-position of the phenoxy ring may serve as a potential reactive site, which could account for its preferential para-metabolism by cytochrome P450 (CYP) (22). In stark contrast, in the present study, the *para*-iodo-nimesulide derivative [^125^I]**1b** exhibited higher stability than the *meta*-iodo-nimesulide derivative [^125^I]**1c**. The differences in metabolic stability observed between our previously reported [^11^C]methoxy-nimesulide derivative and the present [^125^I]iodo-nimesulide derivatives ([^125^I]**1b** and [^125^I]**1c**) suggest that the electronic properties of the substituents may influence their metabolic fate. Similar trends have been reported in previous studies. For example, the *N*-dealkylation of *para*-substituted *N*,*N*-dimethylanilines has been shown to depend on the electron-withdrawing or electron-donating nature of the substituents, which in turn affects the reaction rate (36). Likewise, *para*-substituted azobenzenes with electron-donating groups were reported to promote both CYP binding and reduction reactions in rat liver microsomes (37). These findings support the notion that the introduction of electron-donating substituents at the *para*-position of the benzene ring tends to enhance CYP-mediated metabolism, whereas electron-withdrawing substituents suppress it. However, whether the electronic properties of substituents promote or inhibit CYP-mediated metabolism appears to depend on the specific substrate; it is evident, nonetheless, that these electronic effects play a decisive role. Indeed, computational studies investigating CYP-mediated hydroxylation of aromatic compounds have demonstrated that the electronic characteristics of substituents modulate substrate reactivity, thereby influencing drug metabolism (38). Considering that iodine is an electron-withdrawing substituent, while methoxy is an electron-donating group, these findings support the notion that the electron-donating or electron-withdrawing nature of substituents contributes to the metabolic stability of nimesulide derivatives, as observed in this study. However, further investigation is warranted to clarify this effect.

Perhaps the most significant conceptual advance from this study is the demonstration that the metabolic stability of the nimesulide scaffold can be rationally modulated by the electronic properties of its substituents. Our discovery that the electron-withdrawing *para*-iodo group (**1b**) conferred remarkable *in vivo* stability—in stark contrast to the labile *para*-methoxy group from our previous work (22)—provides a clear and actionable design principle for future radiotracer development based on this scaffold.

Protein binding studies revealed that [^125^I]**1b** binds strongly to plasma proteins, particularly albumin (Table 5). Albumin plays a crucial role in *in vivo* drug delivery (39). The high degree of plasma protein binding observed for [^125^I]**1b** is likely responsible for its prolonged retention in the blood, influencing its overall tissue distribution pattern and representing a key parameter in interpreting its kinetics and uptake in the brain and at sites of inflammation. For effective passage across the BBB, drugs need to be in an unbound or free state rather than bound to plasma proteins (40, 41). Although multiple factors—including lipophilicity, molecular weight, and transporter affinity—contribute to BBB penetration, excessive albumin binding reduces the free fraction of a compound and tends to suppress brain uptake (42, 43). Therefore, the limited brain penetration observed for [^125^I]**1b** is considered to be largely attributable to its high degree of plasma protein binding. Regarding peripheral tissue distribution, several studies on the development of radiolabeled imaging agents targeting tumors have reported correlations between plasma albumin binding and tissue distribution (44–46). These reports consistently indicate that high albumin binding tends to increase blood background levels and slow blood clearance, leading to lower tumor-to-blood ratios at early time points after injection. Over time, however, sustained tumor retention results in a gradual increase in the tumor-to-blood ratio. Importantly, higher albumin binding does not necessarily translate into more favorable tissue distribution; rather, an optimal and reversible degree of albumin binding—often at an intermediate level—appears to be preferable. The distribution of inflammation to sites shows similar behavior to that observed in tumors. In the case of inflammation, however, tracer accumulation is even more strongly influenced by vascular permeability and albumin extravasation than that in tumors, and agents with high albumin binding tend to accumulate more readily at inflammatory sites (47–49). Consistent with this, in the inflammation model, [^125^I]**1b** gradually accumulated in the lesion up to 6 h post-injection, with the inflammation-to-blood ratio increasing slowly from 0.42 ± 0.1 at 30 min to 0.60 ± 0.1 at 6 h. This kinetic profile is likely attributable to its high degree of plasma albumin binding. Nevertheless, it remains unclear whether the exceptionally high albumin binding of [^125^I]**1b** represents the optimal balance for accumulation at inflammatory sites and thus warrants further investigation.

This study has several limitations, yet it also provides valuable insights for future outlook and potential directions. In the course of addressing the three principal challenges outlined for COX-2 tracer development, our work yielded mixed but highly informative results. While we were unable to fully overcome the hurdle of BBB penetration, likely due to the unforeseen issue of high plasma protein binding, our strategy of *para*-iodination successfully solved the critical problem of metabolic stability. Furthermore, we confirmed COX-2-specific binding in the periphery, thus validating the potential of this molecular scaffold for applications outside the CNS. However, several limitations remain that warrant consideration. First, while this evaluation used a single inflammation model, it establishes a strong foundation for future studies. The next logical step will be to assess [^125^I]**1b** in other models of peripheral inflammation, such as arthritis or inflammatory bowel disease, where COX-2 is a key pathological driver. Second, although the metabolic stability of the radiotracers was assessed, detailed identification of all potential metabolites was not performed, leaving uncertainty regarding the complete metabolic pathways. Third, the present findings are based on pre-clinical experiments, and direct extrapolation to human COX-2 imaging should be made with caution. Nevertheless, these limitations do not diminish the overall significance of our results, which provide important insights into the influence of substituent electronic properties on the metabolic fate of nimesulide derivatives. Future studies with larger sample sizes, alternative animal models, and extended metabolic analyses will be essential to further validate and extend these findings.

## 5 Conclusion

In this study, in an effort to address the need for improved COX-2 imaging agents, we synthesized and evaluated radioiodinated derivatives of nimesulide with the aim of developing brain-targeted COX-2 imaging agents. [^125^I]**1b** exhibited high radiochemical purity and remarkable metabolic stability, and its selective accumulation at sites of inflammation was confirmed in a mouse model. In contrast, no clear COX-2–specific accumulation was observed in the brain, and its strong binding to plasma albumin was suggested to be one of the factors limiting brain uptake. These findings indicate that while [^125^I]**1b** has limited potential as a brain-targeted imaging agent at present, it may still hold promise as a valuable radiotracer for visualizing COX-2 expression in peripheral tissues, an important application in its own right. Furthermore, this study demonstrated that the electronic properties of substituents have a marked influence on metabolic stability, providing important insights that can directly inform the future design of imaging agents. Taken together, the present findings offer a solid and informative basis for the rational development of COX-2–targeted molecular imaging agents and highlight a clear path for further investigations using models with higher COX-2 expression and extended metabolic analyses to build upon these foundational results.

## Data Availability

The original contributions presented in the study are included in the article/Supplementary Material, further inquiries can be directed to the corresponding author.

## References

[1] Kam PCA See AUL. Cyclo-oxygenase isoenzymes: physiological and pharmacological role. Anaesthesia. (2000) 55:442–9. 10.1046/j.1365-2044.2000.01271.x 10792135

[2] Bazan NG. COX-2 as a multifunctional neuronal modulator. Nat Med. (2001) 7:414–5. 10.1038/86477 11283664

[3] Minghetti L. Role of COX-2 in inflammatory and degenerative brain diseases. Subcell Biochem. (2007) 42:127–41. 10.1007/1-4020-5688-5_5 17612048

[4] Moussa N Dayoub N. Exploring the role of COX-2 in Alzheimer’s disease: potential therapeutic implications of COX-2 inhibitors. Saudi Pharm J. (2023) 31:101729. 10.1016/j.jsps.2023.101729 37638222 PMC10448476

[5] Kumar A Behl T Jamwal S Kaur I Sood A Kumar P. Exploring the molecular approach of COX and LOX in Alzheimer’s and Parkinson’s disorder. Mol Biol Rep. (2020) 47:9895–912. 10.1007/s11033-020-06033-x 33263931

[6] Prabhakaran J Molotkov A Mintz A Mann JJ. Progress in PET imaging of neuroinflammation targeting COX-2 enzyme. Molecules. (2021) 26:3208. 10.3390/molecules26113208 34071951 PMC8198977

[7] Kenou BV Manly LS Rubovits SB Umeozulu SA Van Buskirk MG Zhang AS Cyclooxygenases as potential PET imaging biomarkers to explore neuroinflammation in dementia. J Nucl Med. (2022) 63(Supplement 1):53S–9. 10.2967/jnumed.121.263199 35649646 PMC9165728

[8] Uzuegbunam BC Rummel C Librizzi D Culmsee C Hooshyar Yousefi BH. Radiotracers for imaging of inflammatory biomarkers TSPO and COX-2 in the brain and in the periphery. Int J Mol Sci. (2023) 24:17419. 10.3390/ijms242417419 38139248 PMC10743508

[9] Yan X Noergaard M Morse CL Liow JS Hong J Greve D PET quantification in healthy humans of cyclooxygenase-2, a potential biomarker of neuroinflammation. J Nucl Med. (2025) 66:398–404. 10.2967/jnumed.124.268525 39848764 PMC11876736

[10] Boyle AJ Narvaez A Tong J Zoghbi SS Pike VW Innis RB Repurposing [^11^C]MC1 for PET imaging of cyclooxygenase-2 in colorectal cancer xenograft mouse models. Mol Imaging Biol. (2022) 24:365–70. 10.1007/s11307-021-01675-0 34766247 PMC9670325

[11] Tietz O Wuest M Marshall A Glubrecht D Hamann I Wang M PET imaging of cyclooxygenase-2 (COX-2) in a pre-clinical colorectal cancer model. EJNMMI Res. (2016) 6:37. 10.1186/s13550-016-0192-9 27112768 PMC4844587

[12] Placzek MS Wilton DK Weïwer M Manter MA Reid SE Meyer CJ A fast-binding, functionally reversible, COX-2 radiotracer for CNS PET imaging. ACS Cent Sci. (2024) 10:1105–14. 10.1021/acscentsci.3c01564 38799654 PMC11117721

[13] Kumar JSD Zanderigo F Prabhakaran J Rubin-Falcone H Parsey RV Mann JJ. *In vivo* evaluation of [^11^C]TMI, a COX-2 selective PET tracer, in baboons. Bioorg Med Chem Lett. (2018) 28:3592–95. 10.1016/j.bmcl.2018.10.049 30396759

[14] Caiazzo E Ialenti A Cicala C. The relatively selective cyclooxygenase-2 inhibitor nimesulide: what’s going on? Eur J Pharmacol. (2019) 848:105–11. 10.1016/j.ejphar.2019.01.044 30689999

[15] Famaey JP. *In vitro* and *in vivo* pharmacological evidence of selective cyclooxygenase-2 inhibition by nimesulide: an overview. Inflamm Res. (1997) 46:437–46. 10.1007/s000110050221 9427063

[16] Shah AA Murray FE Fitzgerald DJ. The *in vivo* assessment of nimesulide cyclooxygenase-2 selectivity. Rheumatology. (1999) 38(Supplement 1):19–23. 10.1093/rheumatology/38.suppl_1.19 10369402

[17] García-Nieto R Pérez C Gago F. Automated docking and molecular dynamics simulations of nimesulide in the cyclooxygenase active site of human prostaglandin-endoperoxide synthase-2 (COX-2). J Comput Aid Mol Des. (2000) 14:147–60. 10.1023/A:1008110924479 10721503

[18] Güngör T Ozleyen A Yılmaz YB Siyah P Ay M Durdağı S New nimesulide derivatives with amide/sulfonamide moieties: selective COX-2 inhibition and antitumor effects. Eur J Med Chem. (2021) 221:113566. 10.1016/j.ejmech.2021.113566 34077833

[19] Wang M Gao M Miller KD Sledge GW Hutchins GD Zheng QH. Radiosynthesis of new carbon-11-labeled nimesulide analogs as potential PET SAER tracers for imaging of aromatase expression in breast cancer. Synth Commun. (2010) 40:749–58. 10.1080/00397910903013747

[20] Sharma N Khanna K Kumar N Karwasra R Janakiraman AK Rajagopal MS. Illuminating insights: clinical study harnessing pharmacoscintigraphy for permeation study of radiolabeled nimesulide topical formulation in healthy human volunteers. Assay Drug Dev Technol. (2023) 21:325–30. 10.1089/adt.2023.053 37801663

[21] Yamamoto Y Hisa T Arai J Saito Y Yamamoto F Mukai T Isomeric methoxy analogs of nimesulide for development of brain cyclooxygense-2 (COX-2)-targeted imaging agents: synthesis, *in vitro* COX-2-inhibitory potency, and cellular transport properties. Bioorg Med Chem. (2015) 23:6807–14. 10.1016/j.bmc.2015.10.007 26455657

[22] Yamamoto Y Tago T Toyohara J Saito Y Yamamoto F. Radiosynthesis and *in vivo* and ex vivo evaluation of isomeric [^11^C]methoxy analogs of nimesulide as brain cyclooxygenase-2-targeted imaging agents. Biol Pharm Bull. (2022) 45:94–103. 10.1248/bpb.b21-00608 34980783

[23] Yamamoto Y. Development of novel COX-2 imaging agents for the diagnosis of brain lesions. Yakugaku Zasshi. (2023) 143:983–7. 10.1248/yakushi.23-00109 38044113

[24] Yamamoto Y Arai J Hisa T Saito Y Mukai T Ohshima T Isomeric iodinated analogs of nimesulide: synthesis, physicochemical characterization, cyclooxygenase-2 inhibitory activity, and transport across Caco-2 cells. Bioorg Med Chem. (2016) 24:3727–33. 10.1016/j.bmc.2016.06.015 27325447

[25] Mee SPH Lee V Baldwin JE. Significant enhancement of the Stille reaction with a new combination of reagents-copper(I) iodide with cesium fluoride. Chemistry. (2005) 11:3294–308. 10.1002/chem.200401162 15786503

[26] Wang C Williams NS. A mass balance approach for calculation of recovery and binding enables the use of ultrafiltration as a rapid method for measurement of plasma protein binding for even highly lipophilic compounds. J Pharm Biomed Anal. (2013) 75:112–7. 10.1016/j.jpba.2012.11.018 23312388 PMC3545278

[27] Sethi PK Muralidhara S Bruckner JV White CA. Measurement of plasma protein and lipoprotein binding of pyrethroids. J Pharmacol Toxicol Methods. (2014) 70:106–11. 10.1016/j.vascn.2014.06.002 24929057

[28] Müller C Farkas R Borgna F Schmid RM Benešová M Schibli R. Synthesis, radiolabeling, and characterization of plasma protein-binding ligands: potential tools for modulation of the pharmacokinetic properties of (radio)pharmaceuticals. Bioconjug Chem. (2017) 28:2372–83. 10.1021/acs.bioconjchem.7b00378 28898054

[29] Duran-Vilaregut J Del Valle J Manich G Camins A Pallàs M Vilaplana J Role of matrix metalloproteinase-9 (MMP-9) in striatal blood-brain barrier disruption in a 3-nitropropionic acid model of Huntington’s disease. Neuropathol Appl Neurobiol. (2011) 37:525–37. 10.1111/j.1365-2990.2010.01157.x 21175737

[30] Villaseñor R Kuennecke B Ozmen L Ammann M Kugler C Grüninger F Region-specific permeability of the blood-brain barrier upon pericyte loss. J Cereb Blood Flow Metab. (2017) 37:3683–94. 10.1177/0271678X17697340 28273726 PMC5718326

[31] Okada K Yamashita U Tsuji S. Cyclooxygenase system contributes to the maintenance of post convulsive period of epileptic phenomena in the genetically epileptic el mice. J UOEH. (2006) 28:265–75. 10.7888/juoeh.28.265 16981403

[32] de Vries EFJ Doorduin J Dierckx RA van Waarde A. Evaluation of [^11^C]rofecoxib as PET tracer for cyclooxygenase 2 overexpression in rat models of inflammation. Nucl Med Biol. (2008) 35:35–42. 10.1016/j.nucmedbio.2007.07.015 18158941

[33] Erfani M Sharifzadeh S Doroudi A Shafiei M. Labeling and evaluation of (99 m) Tc-tricarbonyl-meloxicam as a preferential COX-2 inhibitor for inflammation imaging. J Labelled Comp Radiopharm. (2016) 59:284–90. 10.1002/jlcr.3396 27061432

[34] Küçükgüzel ŞG Küçükgüzel İ Oral B Sezen S Rollas S. Detection of nimesulide metabolites in rat plasma and hepatic subcellular fractions by HPLC-UV/DAD and LC-MS/MS studies. Eur J Drug Metab Pharmacokinet. (2005) 30:127–34. 10.1007/BF03226418 16010872

[35] Carini M Aldini G Stefani R Marinello C Facino RM. Mass spectrometric characterization and HPLC determination of the main urinary metabolites of nimesulide in man. J Pharm Biomed Anal. (1998) 18:201–11. 10.1016/S0731-7085(98)00172-1 9863959

[36] Guengerich FP. Rate-limiting steps in cytochrome P450 catalysis. Biol Chem. (2002) 383:1553–64. 10.1515/BC.2002.175 12452431

[37] Zbaida S Stoddart AM Levine WG. Studies on the mechanism of reduction of azo dye carcinogens by rat liver microsomal cytochrome P-450. Chem Biol Interact. (1989) 69:61–71. 10.1016/0009-2797(89)90099-9 2914330

[38] Bathelt CM Ridder L Mulholland AJ Harvey JN. Mechanism and structure-reactivity relationships for aromatic hydroxylation by cytochrome P450. Org Biomol Chem. (2004) 2:2998–3005. 10.1039/B410729B 15480465

[39] Larsen MT Kuhlmann M Hvam ML Howard KA. Albumin-based drug delivery: harnessing nature to cure disease. Mol Cell Ther. (2016) 4:3. 10.1186/s40591-016-0048-8 26925240 PMC4769556

[40] Pike VW. Considerations in the development of reversibly binding PET radioligands for brain imaging. Curr Med Chem. (2016) 23:1818–69. 10.2174/0929867323666160418114826 27087244 PMC5579844

[41] Aarnio R Alzghool OM Wahlroos S O’Brien-Brown J Kassiou M Solin O Novel plasma protein binding analysis method for a PET tracer and its radiometabolites: a case study with [^11^C]SMW139 to explain the high uptake of radiometabolites in mouse brain. J Pharm Biomed Anal. (2022) 219:114860. 10.1016/j.jpba.2022.114860 35738120

[42] Pike VW. PET radiotracers: crossing the blood-brain barrier and surviving metabolism. Trends Pharmacol Sci. (2009) 30:431–40. 10.1016/j.tips.2009.05.005 19616318 PMC2805092

[43] Amaraneni M Sharma A Pang J Muralidhara S Cummings BS White CA Plasma protein binding limits the blood brain barrier permeation of the pyrethroid insecticide, deltamethrin. Toxicol Lett. (2016) 250–251:21–8. 10.1016/j.toxlet.2016.03.006 27016408

[44] Busslinger SD Becker AE Vaccarin C Deberle LM Renz ML Groehn V Investigations using albumin binders to modify the tissue distribution profile of radiopharmaceuticals exemplified with folate radioconjugates. Cancers (Basel). (2023) 15:4259. 10.3390/cancers15174259 37686538 PMC10486429

[45] Kazuta N Tsuchihashi S Watanabe H Ono M. Fundamental evaluation regarding the relationship between albumin-binding and tumor accumulation of PSMA-targeting radioligands. Ann Nucl Med. (2024) 38:574–83. 10.1007/s12149-024-01930-8 38676906

[46] Krutzek F Donat CK Ullrich M Zarschler K Ludik MC Feldmann A Design and biological evaluation of small-molecule PET-tracers for imaging of programmed death ligand 1. Cancers (Basel). (2023) 15:2638. 10.3390/cancers15092638 37174103 PMC10177516

[47] Sun Z Tong G Liu Y Fan H He W Wang B Dual function of a *in vivo* albumin-labeling tracer for assessment of blood perfusion and vascular permeability in peripheral arterial disease by PET. Front Cardiovasc Med. (2022) 9:738076. 10.3389/fcvm.2022.738076 35211521 PMC8860820

[48] Szade A Grochot-Przeczek A Florczyk U Jozkowicz A Dulak J. Cellular and molecular mechanisms of inflammation-induced angiogenesis. IUBMB Life. (2015) 67:145–59. 10.1002/iub.1358 25899846

[49] Zuckier LS Valdivia AY Zamora E. Does gallium-citrate have yet another story to tell? lessons relevant to the COVID-19 era. Eur J Nucl Med Mol Imaging. (2020) 47:2059–61. 10.1007/s00259-020-04890-z 32468253 PMC7255699

