# Supplementary material for: Radiolabeled para-I-nimesulide: an unexpected tracer for imaging peripheral inflammation
Source: Front Nucl Med. 2026 Jan 2;5:1720380. doi: 10.3389/fnume.2025.1720380 (PMC12808435; doi:10.3389/fnume.2025.1720380)
Supplement: Supplementary file 1 [file Datasheet1.pdf]

YNS-28/SRY16 (b)

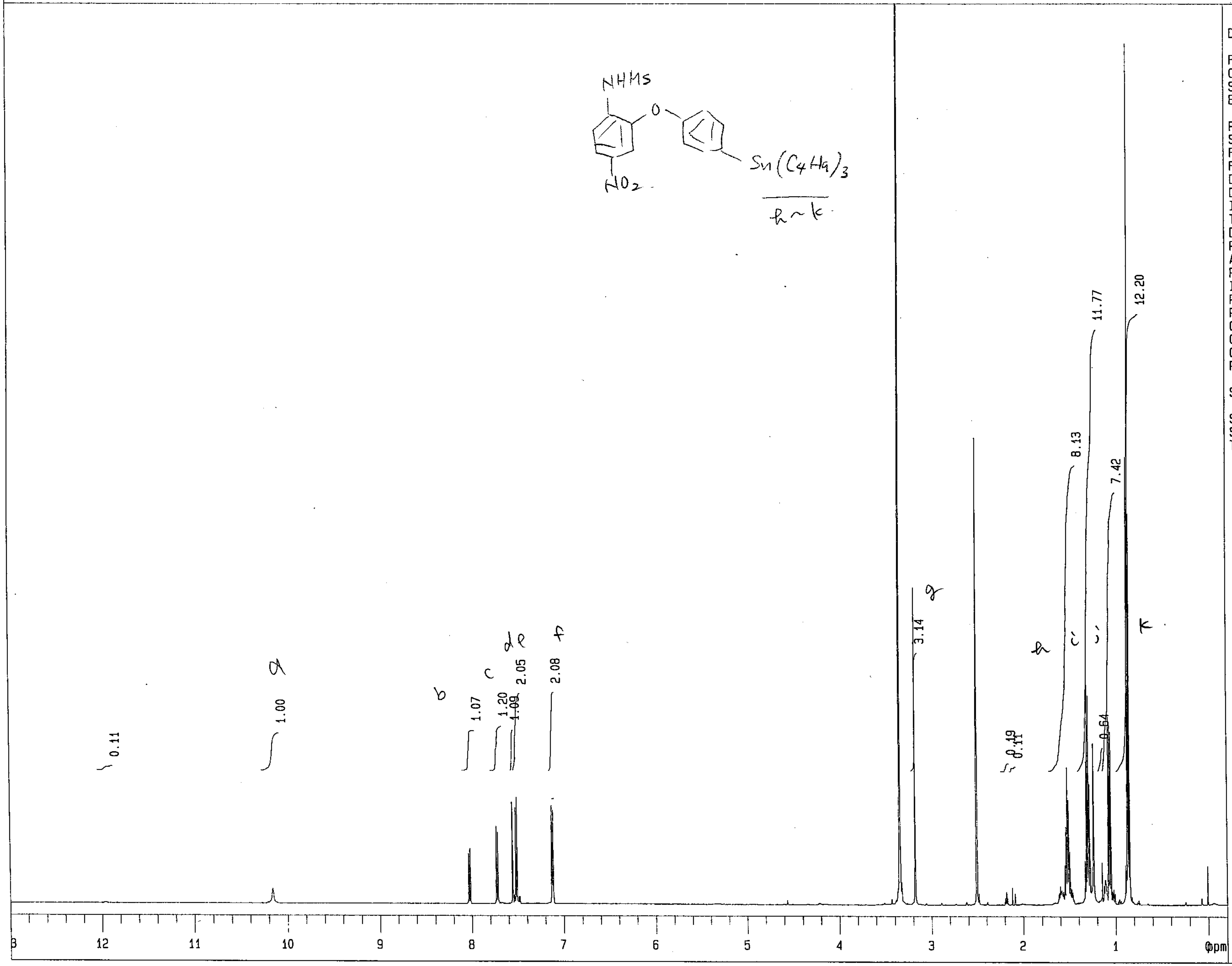


| | | |
|---|---|---|
| Date : Fri Nov 18 11:04:18 2016 | | |
| FileName | : | HSY1H110.nmdata |
| Comment | : | YNS-28/SRY16 (b) |
| SliceHistory | : | |
| EXMODE | : | non |
| POINT | : | 32768 points |
| SAMPO | : | 32768 points |
| FREQU | : | 12004.8 Hz |
| FILTR | : | 6000 Hz |
| DELAY | : | 33.3 usec |
| DEADT | : | 48.3 usec |
| INTVL | : | 83.3 usec |
| TIMES | : | 64 times |
| DUMMY | : | 1 times |
| PD | : | 4.2704 sec |
| ACQTM | : | 2729.5745 msec |
| PREDL | : | 10.00000 msec |
| INIWT | : | 0.5000 msec |
| RESOL | : | 0.37 Hz |
| PW1 | : | 3.45 usec |
| OBNUC | : | 1H |
| OBFRQ | : | 600.05 MHz |
| OBSET | : | 127000.00 Hz |
| RGAIN | : | 16 |
| SCANS | : | 64 times |
| SLVNT | : | DMSO |
| SPINNING | : | 12 Hz |
| TEMP | : | 21.8 C |
