# Supplementary material for: Radiolabeled para-I-nimesulide: an unexpected tracer for imaging peripheral inflammation
Source: Front Nucl Med. 2026 Jan 2;5:1720380. doi: 10.3389/fnume.2025.1720380 (PMC12808435; doi:10.3389/fnume.2025.1720380)
Supplement: Supplementary file 2 [file Datasheet2.pdf]

YNS-45/SRY45

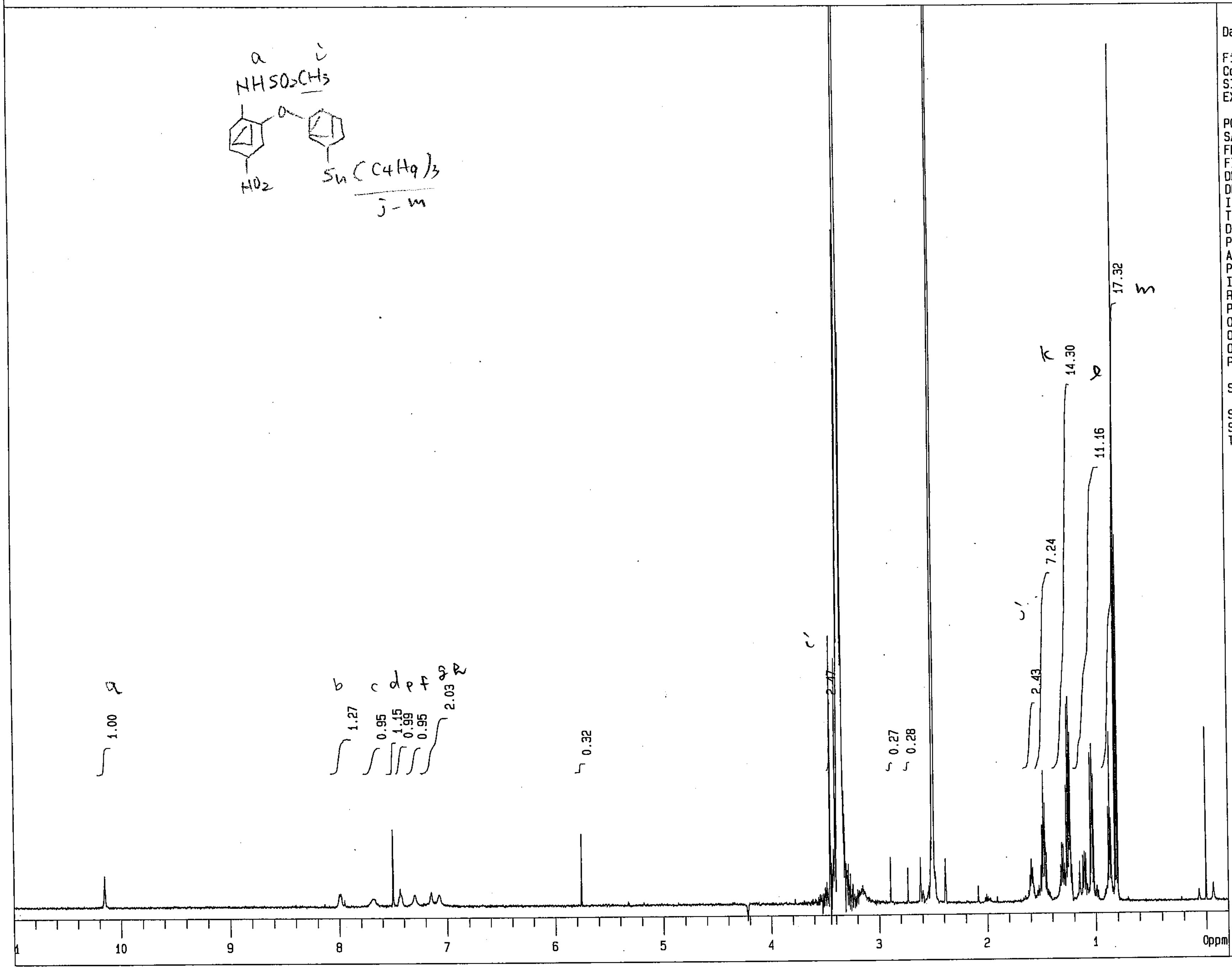

Date : Tue May 2 17:52:41 2017

FileName	:	HSY1H114.nmdata
Comment	:	YNS-45/SRY45
SliceHistory	:	
EXMODE	:	non
POINT	:	32768 points
SAMPO	:	32768 points
FREQU	:	12004.8 Hz
FILTR	:	6000 Hz
DELAY	:	33.3 usec
DEADT	:	48.3 usec
INTVL	:	83.3 usec
TIMES	:	64 times
DUMMY	:	1 times
PD	:	4.2704 sec
ACQTM	:	2729.5745 msec
PREDL	:	10.00000 msec
INIWT	:	0.5000 msec
RESOL	:	0.37 Hz
PW1	:	3.45 usec
OBNUC	:	1H
OBFRQ	:	600.05 MHz
OBSET	:	127000.00 Hz
RGAIN	:	20
SCANS	:	64 times
SLVNT	:	DMSO
SPINNING	:	13 Hz
TEMP	:	20.0 C
