# Supplementary material for: Radiolabeled para-I-nimesulide: an unexpected tracer for imaging peripheral inflammation
Source: Front Nucl Med. 2026 Jan 2;5:1720380. doi: 10.3389/fnume.2025.1720380 (PMC12808435; doi:10.3389/fnume.2025.1720380)
Supplement: Supplementary file 3 [file Datasheet3.pdf]

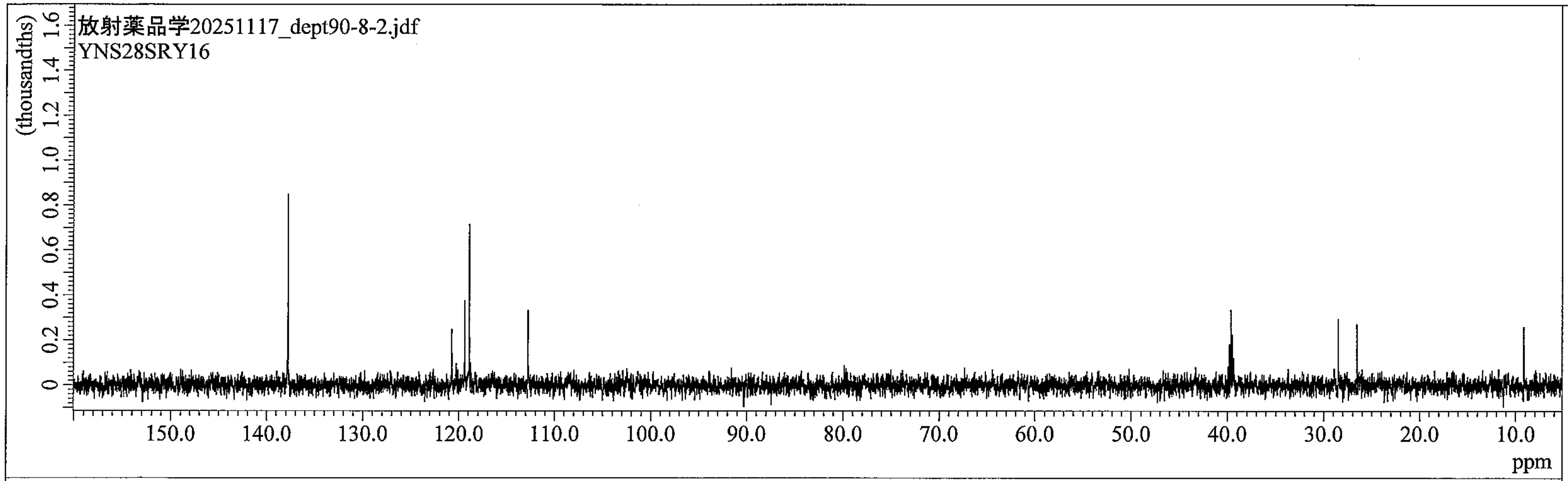

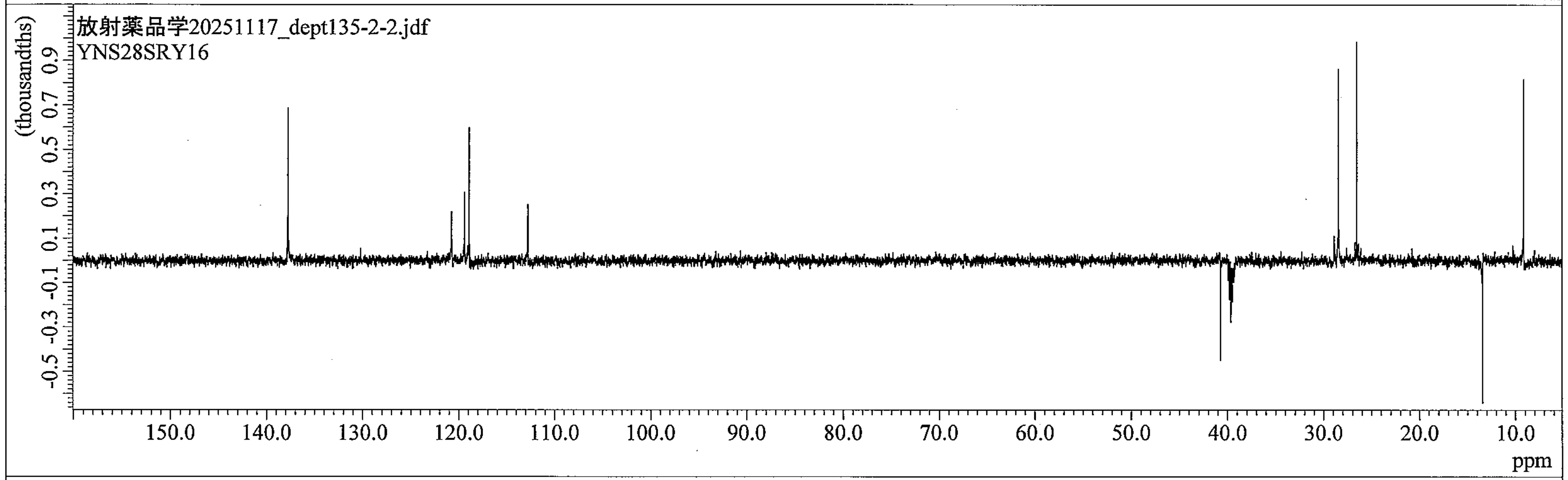

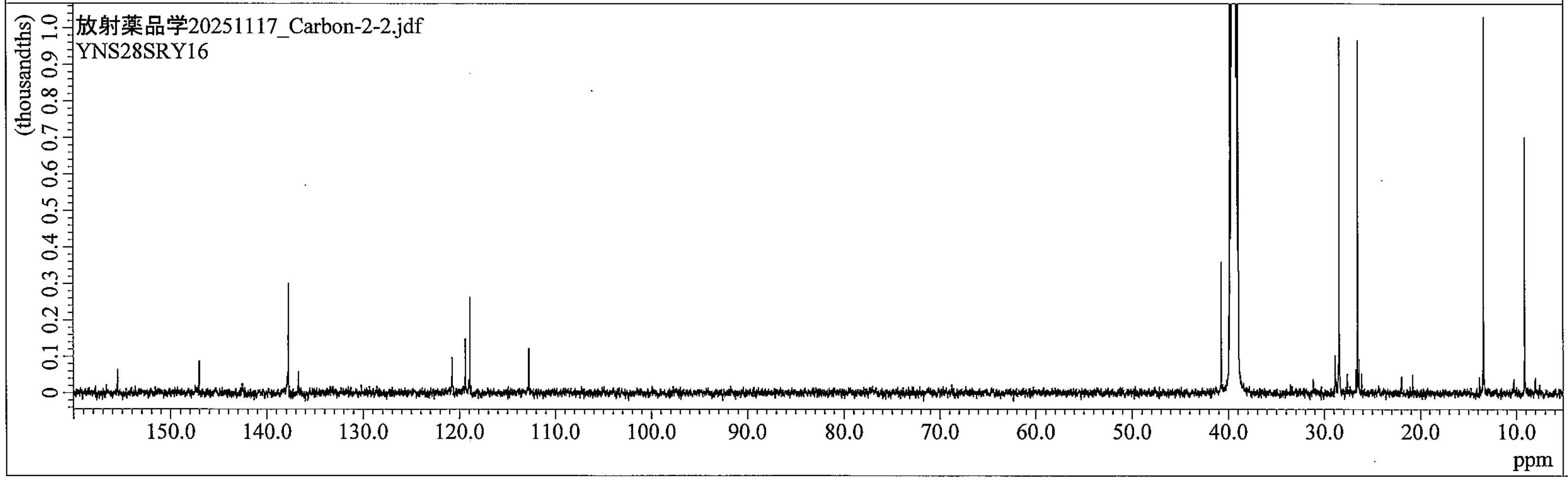

Filename	= 放射薬品学20251117_Carb
Author	= delta
Experiment	= carbon.jxp
Sample_Id	= 放射薬品学20251117
Solvent	= DMSO-D6
Actual_Start_Time	= 17-NOV-2025 17:12:
Revision_Time	= 18-NOV-2025 08:45:
Comment	= YNS28SRY16
Data_Format	= 1D REAL
Dim_Size	= 26214
X_Domain	= Carbon13
Dim_Title	= Carbon13
Dim_Units	= [ppm]
Dimensions	= X
Spectrometer	= JNM-ECZ600R/S1
Field_Strength	= 14.09636928[T] (60
X_Acq_Duration	= 0.69206016[s]
X_Domain	= Carbon13
X_Freq	= 150.91343039[MHz]
X_Offset	= 100[ppm]
X_Points	= 32768
X_Prescans	= 4
X_Resolution	= 1.44496109[Hz]
X_Sweep	= 47.34848485[kHz]
X_Sweep_Clipped	= 37.87878788[kHz]
Irr_Domain	= Proton
Irr_Freq	= 600.1723046[MHz]
Irr_Offset	= 5[ppm]
Blanking	= 2.0[us]
Clipped	= FALSE
Scans	= 8000
Total_Scans	= 8000
Relaxation_Delay	= 2[s]
Recvr_Gain	= 40
Temp_Get	= 23.4[dC]
X_90_Width	= 13.4[us]
X_Acq_Time	= 0.69206016[s]
X_Angle	= 30[deg]
X_Atn	= 9.5[dB]
X_Pulse	= 4.46666667[us]
Irr_Atn_Dec	= 24.84[dB]
Irr_Atn_Dec_Calc	= 24.84[dB]
Irr_Atn_Dec_Default_Calc	= 24.84[dB]
Irr_Atn_Noe	= 24.84[dB]
Irr_Dec_Bandwidth_Hz	= 7.23684211[kHz]
Irr_Dec_Bandwidth_Ppm	= 12.05794078[ppm]
Irr_Dec_Freq	= 600.1723046[MHz]
Irr_Dec_Merit_Factor	= 2.2
Irr_Decoupling	= TRUE
Irr_Noe	= TRUE
Irr_Noise	= WALTZ
Irr_Offset_Default	= 5[ppm]
Irr_Pwidth	= 76[us]
Irr_Pwidth_Default	= 76[us]
Irr_Pwidth_Default_Calc	= 76[us]
Irr_Pwidth_Templ	= 76[us]
Irr_Wurst	= FALSE
Decimation_Rate	= 0
Experiment_Path	= c:\Program Files\J
Initial_Wait	= 1[s]
Noe_Time	= 2[s]
Noe_Time_Flag	= FALSE
Relaxation_Delay_Calc	= 0[s]
Relaxation_Delay_Temp	= 2[s]
Repetition_Time	= 2.69206016[s]
