# Supplementary figures and images for: Radiolabeled para-I-nimesulide: an unexpected tracer for imaging peripheral inflammation
Source: Front Nucl Med. 2026 Jan 2;5:1720380. doi: 10.3389/fnume.2025.1720380 (PMC12808435; doi:10.3389/fnume.2025.1720380)

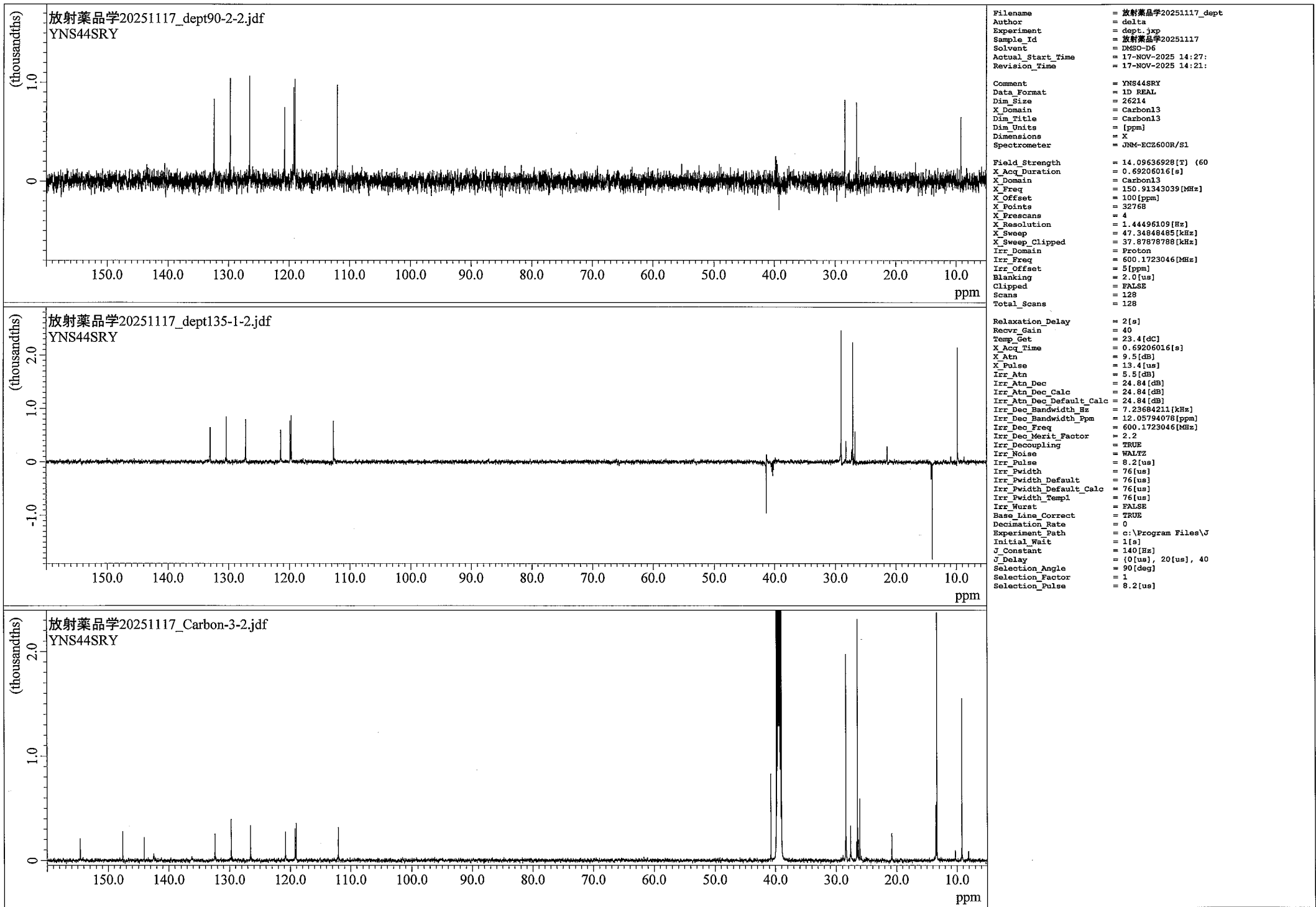

Supplement: Supplementary file 4 [file Datasheet4.pdf]
