# Supplementary material for: Radiolabeled para-I-nimesulide: an unexpected tracer for imaging peripheral inflammation
Source: Front Nucl Med. 2026 Jan 2;5:1720380. doi: 10.3389/fnume.2025.1720380 (PMC12808435; doi:10.3389/fnume.2025.1720380)
Supplement: Supplementary file 5 [file Table1.pdf]

Supplementary Material

Supplementary Tables

Table S1A Biodistribution study of [^{125}I]**1b** in BALB/c mice

Tissue	Uptake (%ID/g)[a)]					
	30 min	1 h	2 h	6 h	12 h	24 h
Blood	13.99 ± 3.7	12.39 ± 1.9	11.48 ± 2.2	12.62 ± 2.1	10.72 ± 1.7	7.82 ± 1.2
Heart	6.59 ± 2.5	5.16 ± 0.5	4.72 ± 1.1	4.64 ± 0.8	4.04 ± 0.3	2.71 ± 0.2
Lung	8.46 ± 2.6	10.01 ± 2.0	8.65 ± 2.3	8.27 ± 2.1	8.68 ± 1.1	4.37 ± 0.7
Liver	9.30 ± 3.6	8.71 ± 1.0	8.07 ± 1.1	7.76 ± 1.0	6.24 ± 0.9	4.60 ± 0.8
Pancreas	4.97 ± 2.3	3.30 ± 0.7	3.11 ± 0.6	3.19 ± 0.3	2.63 ± 0.2	1.66 ± 0.3
Spreen	3.79 ± 1.8	3.34 ± 0.7	2.94 ± 0.6	2.76 ± 0.3	2.45 ± 0.2	1.68 ± 0.2
Kidney	6.24 ± 3.5	5.24 ± 0.6	5.16 ± 0.9	4.99 ± 0.3	4.29 ± 0.6	3.56 ± 0.5
Stomach	3.24 ± 1.0	3.56 ± 0.7	3.48 ± 0.7	4.05 ± 1.1	1.82 ± 0.4	2.12 ± 0.6
S.intestine	7.43 ± 5.6	6.38 ± 1.4	5.68 ± 1.5	4.69 ± 1.0	4.31 ± 1.0	3.55 ± 1.0
Muscle	2.56 ± 0.8	5.63 ± 4.3	4.71 ± 3.2	2.97 ± 1.7	2.17 ± 0.8	1.53 ± 0.4
Thyroid[b)]	0.09 ± 0.0	0.08 ± 0.0	0.06 ± 0.0	0.08 ± 0.0	0.09 ± 0.0	0.10 ± 0.0
Brain	0.89 ± 0.3	1.00 ± 0.2	1.16 ± 0.2	1.14 ± 0.3	1.00 ± 0.1	0.47 ± 0.1

[a)] Data are presented as percent injected dose per gram of tissue (mean ± standard deviation). Sample sizes were n = 4 for 30 min; n = 5 for 1 h and 6 h; n = 6 for 12 h and 24 h; and n = 8 for 2 h. [b)] Data are presented as percent injected dose per organ.

Table S1B Biodistribution study of [^{125}I]**1c** in BALB/c mice

Tissue	Uptake (%ID/g)[a)]					
	30 min	1 h	2 h	6 h	12 h	24 h
Blood	10.59 ± 2.0	8.58 ± 1.0	6.99 ± 1.5	3.09 ± 0.6	0.62 ± 0.1	0.14 ± 0.0
Heart	9.04 ± 3.8	4.26 ± 0.9	2.94 ± 0.5	1.59 ± 0.3	0.30 ± 0.1	0.05 ± 0.0
Lung	8.09 ± 2.5	5.52 ± 1.2	3.97 ± 1.0	2.08 ± 0.5	0.48 ± 0.1	0.09 ± 0.0
Liver	8.35 ± 1.9	8.85 ± 1.3	7.60 ± 1.3	6.68 ± 2.2	1.00 ± 0.2	0.41 ± 0.1
Pancreas	5.45 ± 1.7	2.81 ± 0.8	2.41 ± 0.5	1.24 ± 0.2	0.26 ± 0.1	0.03 ± 0.0
Spreen	5.77 ± 3.1	2.46 ± 0.8	1.64 ± 0.4	0.87 ± 0.2	0.14 ± 0.0	0.03 ± 0.0
Kidney	6.57 ± 1.2	5.60 ± 0.7	4.30 ± 1.0	2.55 ± 0.5	0.48 ± 0.1	0.12 ± 0.0
Stomach	5.74 ± 2.3	5.65 ± 1.2	6.47 ± 3.6	5.84 ± 3.6	1.47 ± 1.2	0.27 ± 0.1
S.intestine	8.99 ± 5.1	9.96 ± 4.1	10.57 ± 9.3	11.74 ± 14.9	0.80 ± 0.4	0.11 ± 0.0
Muscle	7.72 ± 4.1	2.33 ± 0.7	1.59 ± 0.3	1.66 ± 1.2	0.16 ± 0.0	0.04 ± 0.0
Thyroid[b)]	0.05 ± 0.0	0.05 ± 0.0	0.10 ± 0.0	0.43 ± 0.1	0.42 ± 0.2	0.49 ± 0.1
Brain	1.30 ± 0.1	1.38 ± 0.3	1.27 ± 0.2	0.51 ± 0.1	0.11 ± 0.0	0.01 ± 0.0

[a)] Data are presented as percent injected dose per gram of tissue (mean ± standard deviation). Sample sizes were n = 5 for 12 h; n = 6 for 2 h, 6 h and 24 h; n = 7 for 30 min; and n = 9 for 1 h. [b)] Data are presented as percent injected dose per organ.

Table S2A Biodistribution study of $[^{125}I]$**1b** in a mouse model of inflammation

Tissue	Uptake (%ID/g)[b)]					
	30 min	1 h	2 h	6 h	12 h	24 h
Blood	11.74 ± 2.8	14.52 ± 1.6	14.07 ± 2.7	12.65 ± 1.9	9.33 ± 1.6	9.00 ± 1.2
Heart	5.06 ± 1.2	5.85 ± 0.6	5.89 ± 1.4	4.92 ± 1.1	3.97 ± 0.7	3.82 ± 0.5
Lung	7.69 ± 2.2	8.48 ± 1.3	8.97 ± 2.6	8.00 ± 2.8	4.92 ± 0.9	4.63 ± 0.9
Liver	7.87 ± 2.5	10.65 ± 0.8	10.33 ± 2.6	9.05 ± 1.4	5.60 ± 0.6	5.59 ± 1.3
Pancreas	3.48 ± 1.1	3.99 ± 0.2	4.38 ± 1.5	3.83 ± 0.8	2.32 ± 0.4	1.92 ± 0.3
Spreen	2.63 ± 0.8	3.26 ± 0.3	3.71 ± 1.0	2.92 ± 0.6	1.84 ± 0.2	1.77 ± 0.3
Kidney	5.03 ± 1.5	6.93 ± 0.7	6.66 ± 1.7	6.04 ± 1.1	3.48 ± 0.8	3.90 ± 0.7
Stomach	3.22 ± 1.0	3.69 ± 0.4	3.84 ± 1.3	3.21 ± 0.4	1.94 ± 0.8	2.03 ± 0.2
S.intestine	4.45 ± 1.1	5.13 ± 1.1	6.33 ± 1.9	4.55 ± 1.1	2.83 ± 0.2	2.88 ± 0.4
Muscle	2.75 ± 1.5	2.31 ± 0.6	2.53 ± 0.2	1.93 ± 0.4	1.44 ± 0.4	1.37 ± 0.3
Inflammation	4.97 ± 1.4	5.79 ± 1.7	7.20 ± 1.9	7.54 ± 1.6	4.29 ± 0.7	4.81 ± 1.0
Thyroid[b)]	0.08 ± 0.0	0.08 ± 0.0	0.08 ± 0.0	0.10 ± 0.0	0.09 ± 0.1	0.14 ± 0.0
Brain	0.96 ± 0.3	1.28 ± 0.1	1.38 ± 0.5	1.10 ± 0.3	0.59 ± 0.1	0.64 ± 0.1

[a)] Data are presented as percent injected dose per gram of tissue (mean ± standard deviation). Sample sizes were n = 6 for 1 h, 2 h and 24 h; n = 7 for 6 h and 12 h; and n = 10 for 30 min. [b)] Data are presented as percent injected dose per organ.

Table S2B Biodistribution study of [^{125}I]**1c** in a mouse model of inflammation

Tissue	Uptake (%ID/g)[b]					
	30 min	1 h	2 h	6 h	12 h	24 h
Blood	10.59 ± 2.3	8.10 ± 3.5	8.19 ± 0.6	4.18 ± 0.5	1.44 ± 0.3	0.19 ± 0.1
Heart	7.20 ± 2.4	6.28 ± 4.2	5.14 ± 0.5	2.47 ± 0.5	2.59 ± 1.0	0.10 ± 0.0
Lung	8.27 ± 2.1	5.17 ± 2.3	5.43 ± 1.5	2.48 ± 0.3	3.81 ± 2.1	0.12 ± 0.0
Liver	10.90 ± 2.5	8.51 ± 4.1	9.31 ± 0.9	5.67 ± 0.7	3.30 ± 1.2	0.55 ± 0.1
Pancreas	6.00 ± 2.3	4.16 ± 1.5	4.47 ± 1.3	1.76 ± 0.2	1.57 ± 0.6	0.07 ± 0.0
Spreen	3.57 ± 1.9	2.01 ± 0.9	2.35 ± 0.6	1.21 ± 0.2	0.99 ± 0.5	0.05 ± 0.0
Kidney	8.20 ± 1.2	6.19 ± 2.2	6.88 ± 0.4	3.92 ± 0.5	2.64 ± 0.9	0.20 ± 0.1
Stomach	5.90 ± 1.7	4.69 ± 1.7	6.38 ± 1.4	4.21 ± 1.4	1.46 ± 0.7	0.17 ± 0.1
S.intestine	10.94 ± 4.0	6.97 ± 3.7	12.04 ± 7.6	3.56 ± 1.8	4.29 ± 2.6	0.21 ± 0.2
Muscle	2.40 ± 0.5	1.88 ± 0.9	1.81 ± 0.4	0.94 ± 0.4	0.79 ± 0.3	0.03 ± 0.0
Inflammation	6.13 ± 1.1	5.17 ± 2.6	5.04 ± 0.9	2.88 ± 0.3	2.40 ± 1.0	0.21 ± 0.1
Thyroid[b]	0.11 ± 0.0	0.09 ± 0.0	0.16 ± 0.0	0.50 ± 0.1	0.32 ± 0.1	0.34 ± 0.1
Brain	2.06 ± 0.5	1.51 ± 0.6	1.89 ± 0.2	0.72 ± 0.1	0.22 ± 0.1	0.03 ± 0.0

[a] Data are presented as percent injected dose per gram of tissue (mean ± standard deviation). Sample sizes were n = 6 for 30 min, 2 h, 6 h and 24h; and n = 7 for 1 h and 12 h. [b] Data are presented as percent injected dose per organ.

Table S3A Biodistribution study of [^{125}I]**1b** with or without cyclooxygenase inhibitors 6 h after injection in a mouse model of inflammation

Tissue	Uptake (%ID/g)[b)]				
	Control	Nimesulide	**1b**	Indomethacin	Celecoxib
Blood	12.96 ± 2.3	12.06 ± 0.5	13.14 ± 3.2	16.48 ± 0.6	14.39 ± 1.5
Heart	5.69 ± 1.0	4.80 ± 0.6	5.85 ± 1.8	6.67 ± 0.7	6.31 ± 0.4
Lung	8.01 ± 2.7	9.39 ± 3.9	11.29 ± 3.4	10.21 ± 2.5	10.57 ± 1.5
Liver	7.36 ± 1.2	7.16 ± 0.2	7.25 ± 1.2	8.12 ± 0.5	8.26 ± 0.7
Pancreas	2.86 ± 0.4	2.73 ± 0.2	2.81 ± 0.7	3.58 ± 0.3	3.23 ± 0.3
Spreen	2.85 ± 0.5	2.87 ± 0.2	2.55 ± 0.4	2.53 ± 0.1	3.16 ± 0.3
Kidney	5.17 ± 0.8	4.66 ± 0.4	4.96 ± 1.1	5.37 ± 0.4	5.56 ± 0.8
Stomach	3.01 ± 0.9	2.56 ± 0.2	2.46 ± 0.7	2.61 ± 0.4	3.15 ± 0.4
S.intestine	4.65 ± 0.9	4.33 ± 0.2	4.61 ± 1.2	4.57 ± 1.0	5.35 ± 0.7
Muscle	2.04 ± 0.5	2.88 ± 0.4	2.88 ± 0.7	2.78 ± 0.8	3.82 ± 0.9
Inflammation	6.25 ± 1.0	5.82 ± 1.3	7.09 ± 2.1	6.52 ± 0.8	6.45 ± 1.1
Thyroid[b)]	0.10 ± 0.0	0.09 ± 0.0	0.10 ± 0.0	0.12 ± 0.1	0.07 ± 0.0
Brain	1.14 ± 0.3	0.99 ± 0.1	1.28 ± 0.6	1.32 ± 0.2	1.23 ± 0.2

[a)] Data are presented as percent injected dose per gram of tissue (mean ± standard deviation). Sample sizes were n = 4 for nimesulide and celecoxib, n = 5 for indomethacin, and n = 6 for **1b** and control.
[b)] Data are presented as percent injected dose per organ.

Table S4 Determination of protein binding percentages for [^{14}C]diazepam, [^{14}C]antipyrine, and [^{125}I]**1b**

	Percentage of protein binding (%)			
	[^{14}C]Diazepam	[^{3}H]Cyclosporin A	[^{14}C]Antipyrine	[^{125}I]**1b**
Human plasma	99.2 ± 0.0	98.2 ± 0.1	41.2 ± 1.4	99.8 ± 0.1
Mouse plasma	97.6 ± 0.2	98.1 ± 0.1	36.4 ± 6.5	99.8 ± 0.0
Human albumin	99.1 ± 0.1	97.6 ± 0.1	28.9 ± 0.7	99.6 ± 0.0
Human α1-acid glycoprotein	95.1 ± 0.5	96.8 ± 0.1	18.9 ± 0.8	98.1 ± 0.2

Protein binding percentages were determined using Amicon devices (n = 3).

Supplementary Figures

Figure S1A ^{1}H NMR Spectrum of Compound **2b**

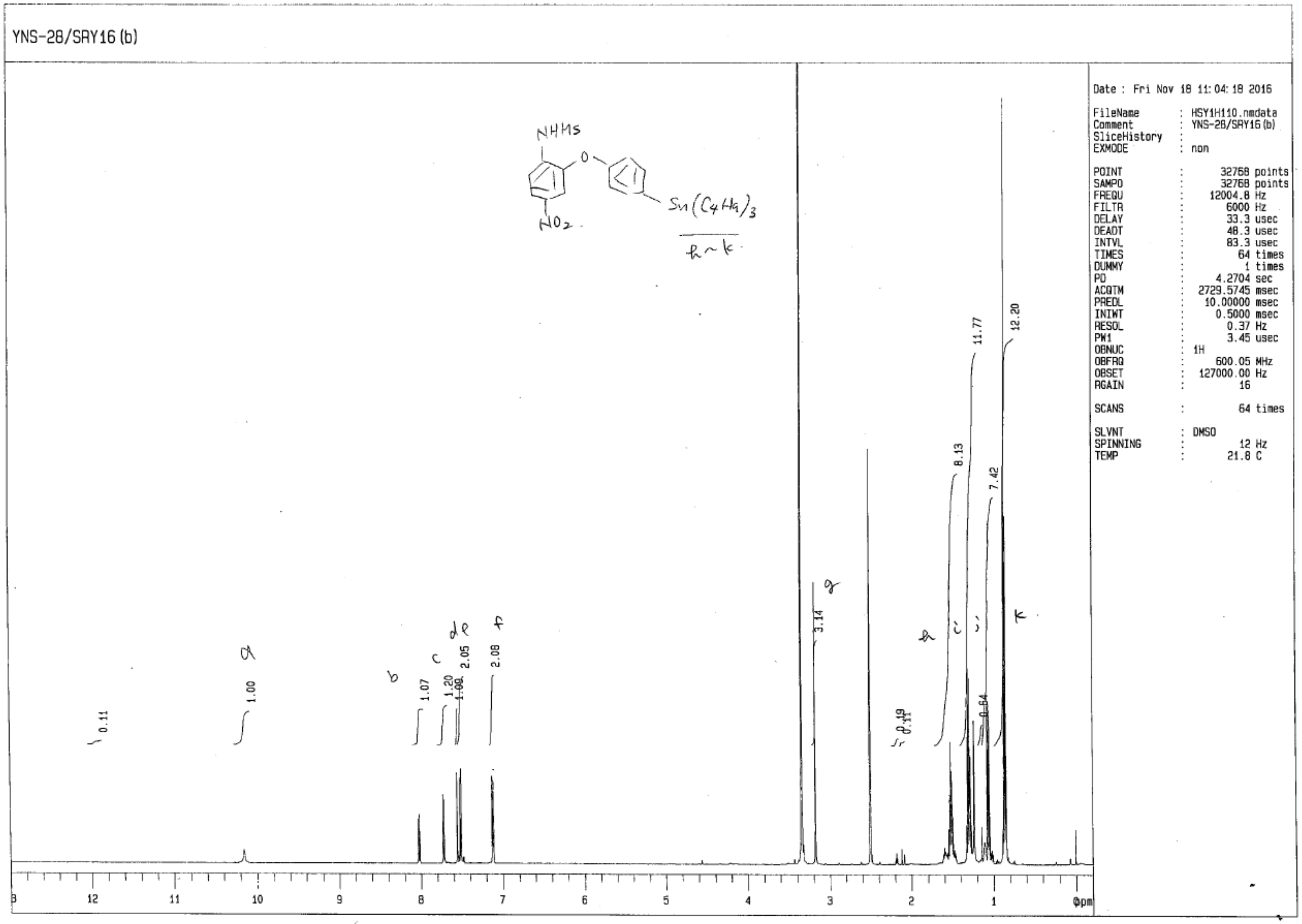

Figure S1B ^{1}H NMR Spectrum of Compound **2c**

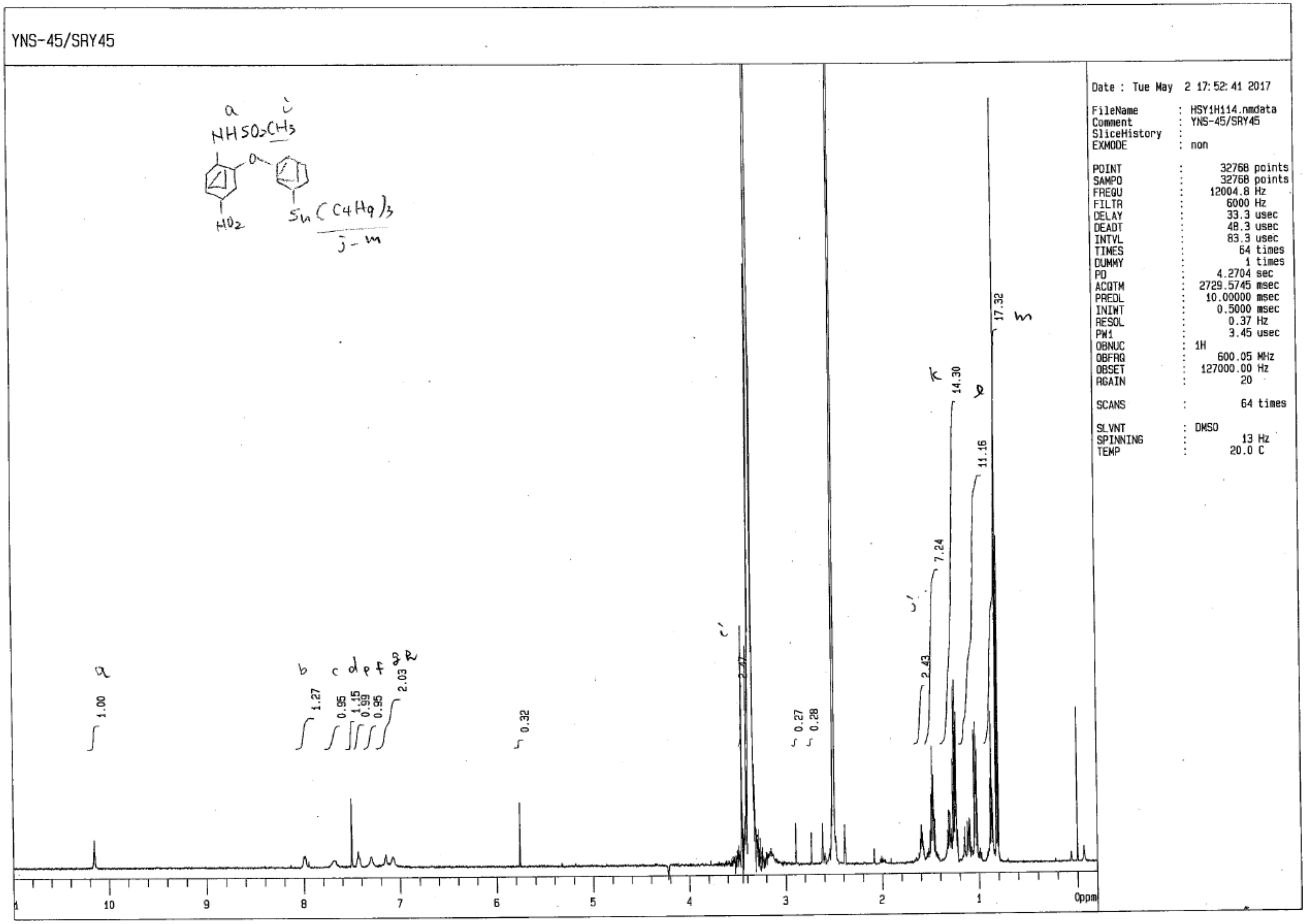

Figure S1C ^{13}C NMR Spectrum of Compound **2b**

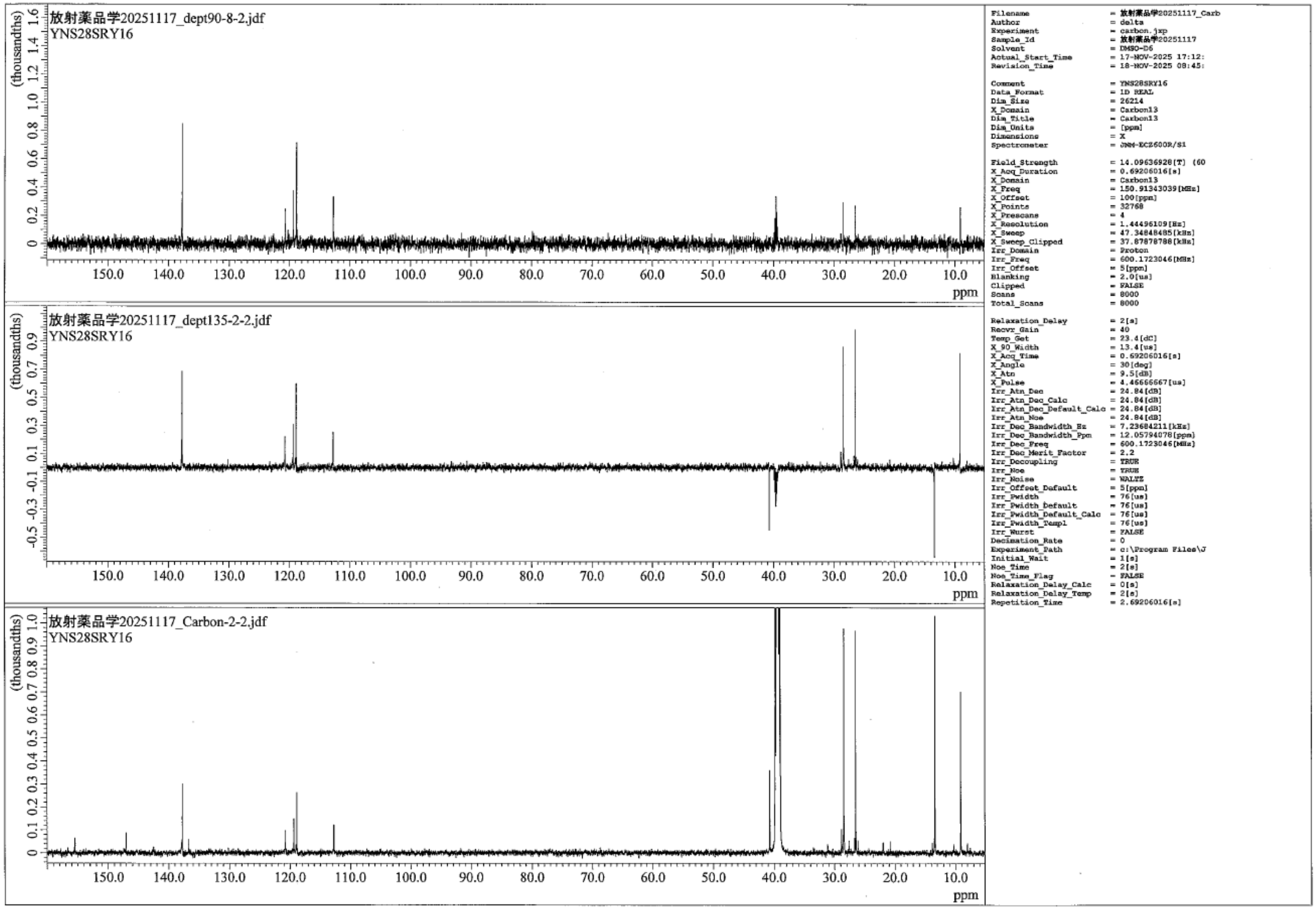

Figure S1D ^{13}C NMR Spectrum of Compound **2c**

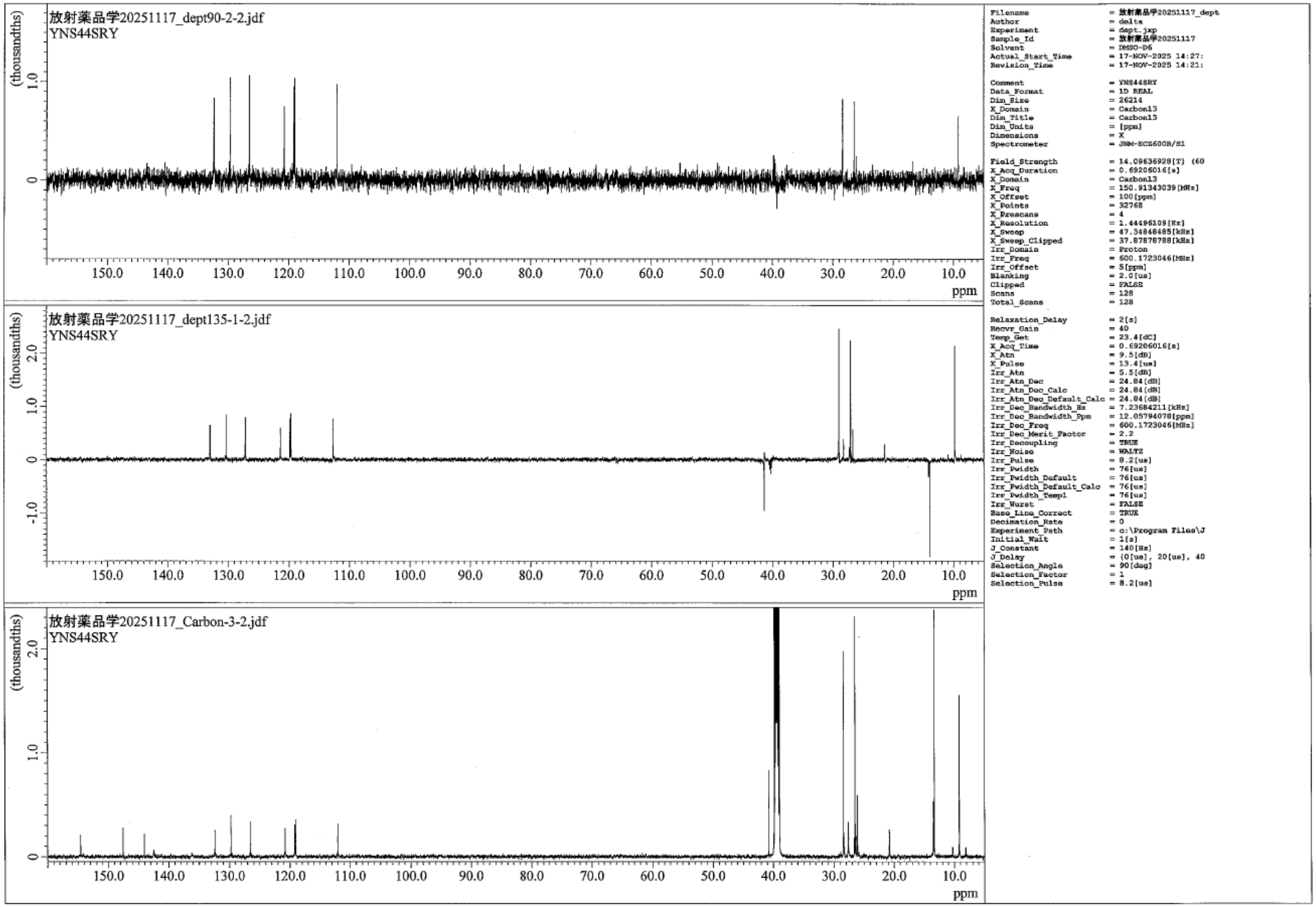

Figure S2A Autoradiography images of coronal brain sections after administration of [^{125}I]**1b**.

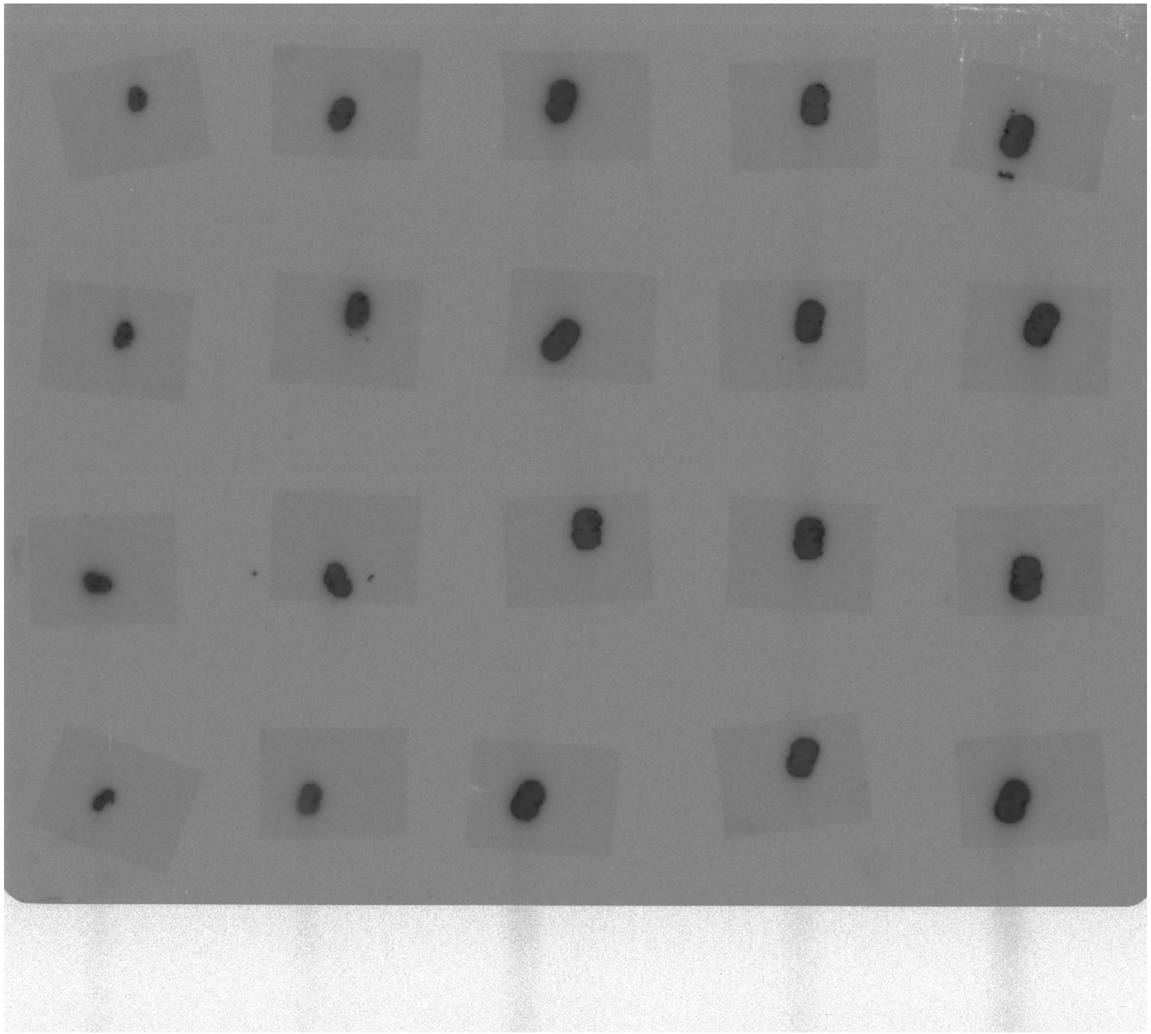

Representative autoradiograms of coronal brain sections obtained at 30 min, 1 h, 2 h, and 3 h after administration of [^{125}I]**1b**. Rows correspond to different time points (from top to bottom: 1 h, 2 h, 3 h, and 30 min). Columns correspond to different coronal levels (from left to right: bregma A1.0 mm, 0.0 mm, P1.4 mm, P2.0 mm, and P2.1–2.2 mm). Images are shown prior to trimming.

Figure S2B Autoradiography images of coronal brain sections after administration of [^{125}I]**1b**

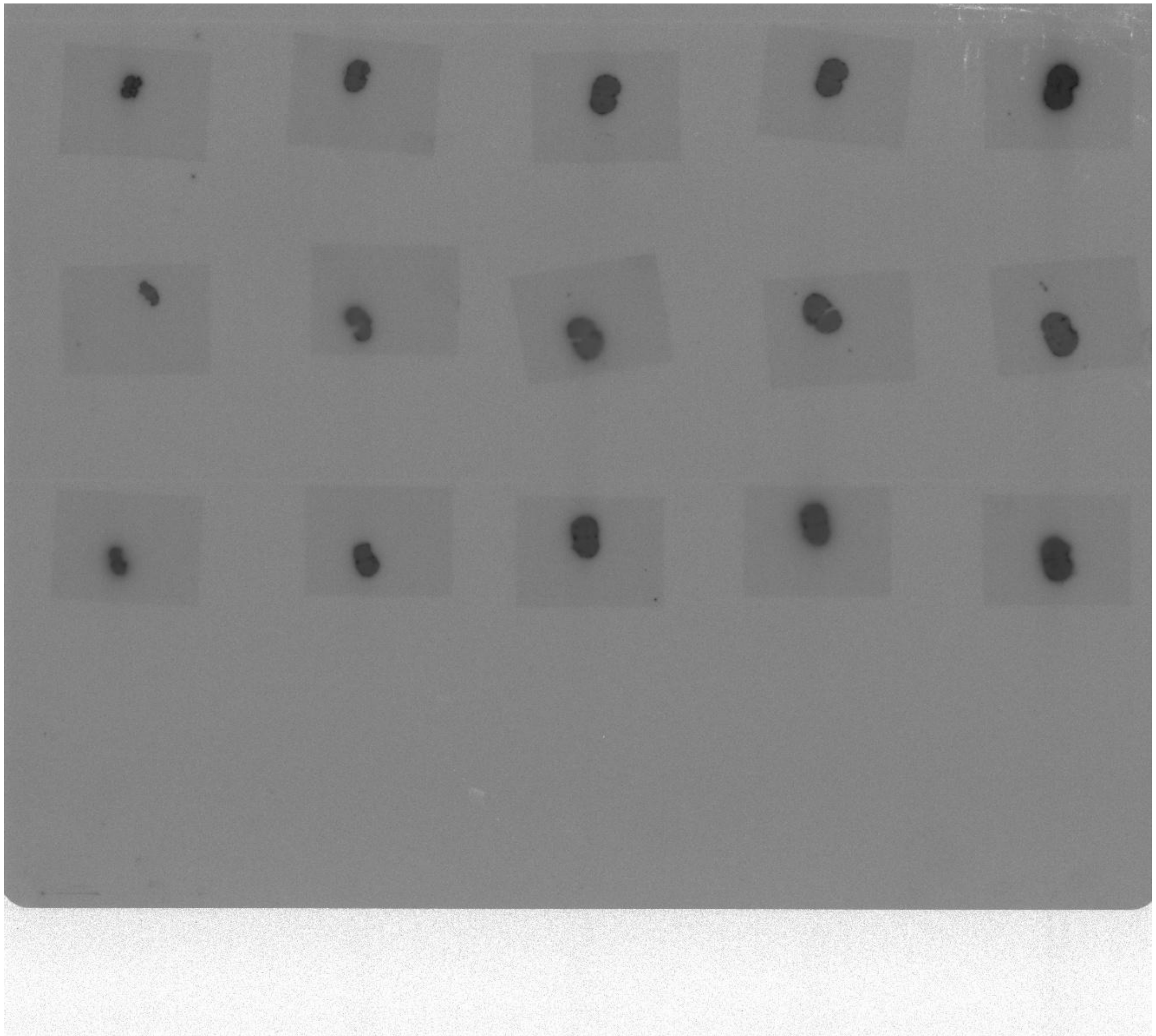

Representative autoradiograms of coronal brain sections obtained at 6 h, 12 h, and 24 h after administration of [^{125}I]**1b**. Rows correspond to different time points (from top to bottom: 12 h, 24 h, and 6 h). Columns correspond to different coronal levels (from left to right: bregma A1.0 mm, 0.0 mm, P1.4 mm [two adjacent sections], and P2.0 mm). Images are shown prior to trimming.

Figure S2C Autoradiography images of coronal brain sections after administration of [^{125}I]**1c**.

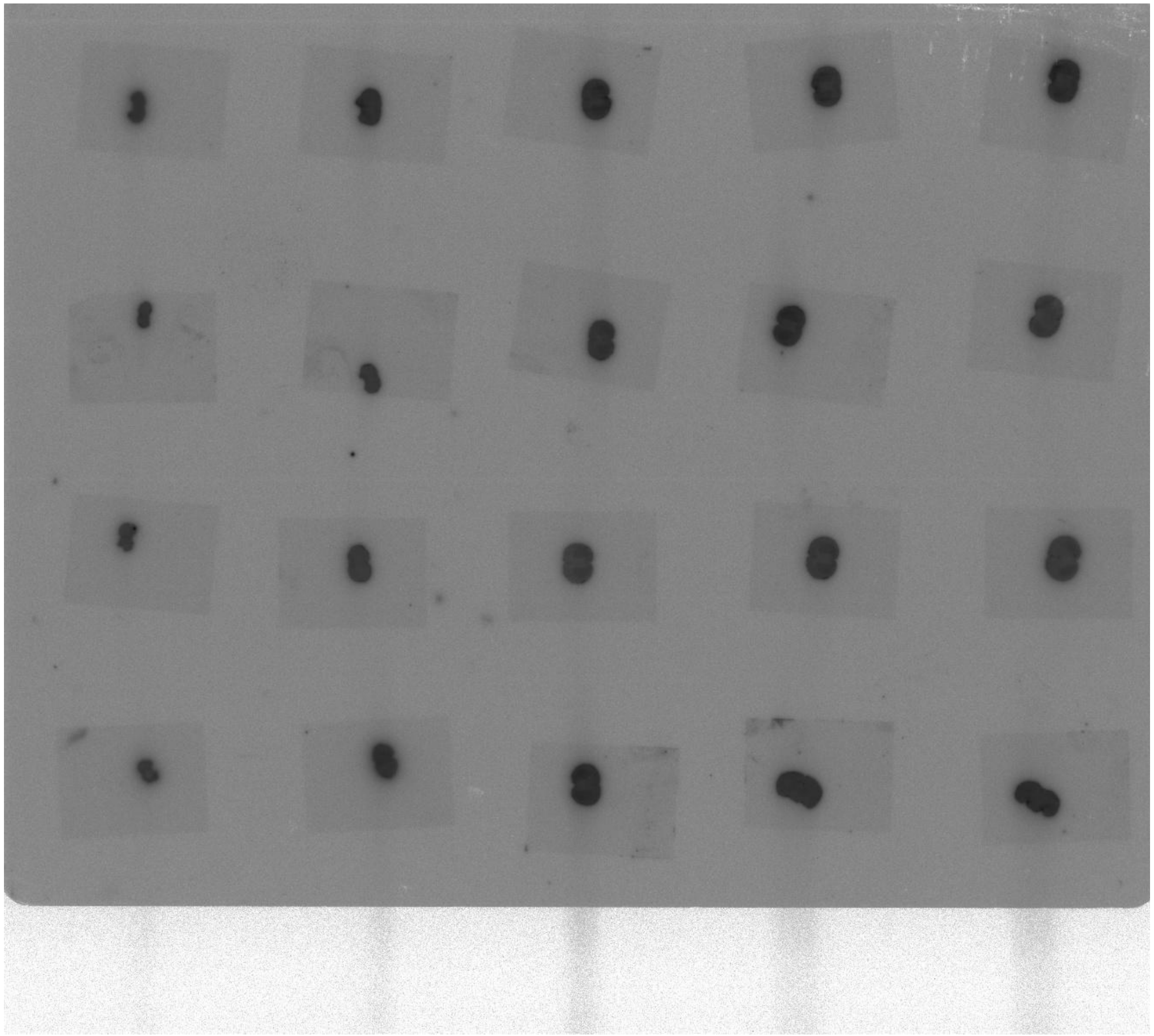

Representative autoradiograms of coronal brain sections obtained at 30 min, 1 h, 2 h, and 3 h after administration of [^{125}I]**1c**. Rows correspond to different time points (from top to bottom: 1 h, 2 h, 3 h, and 30 min). Columns correspond to different coronal levels (from left to right: bregma A1.0 mm, 0.0 mm, P1.4 mm, P2.0 mm, and P2.1–2.2 mm). Images are shown prior to trimming.

Figure S2D Autoradiography images of coronal brain sections after administration of [^{125}I]**1c**.

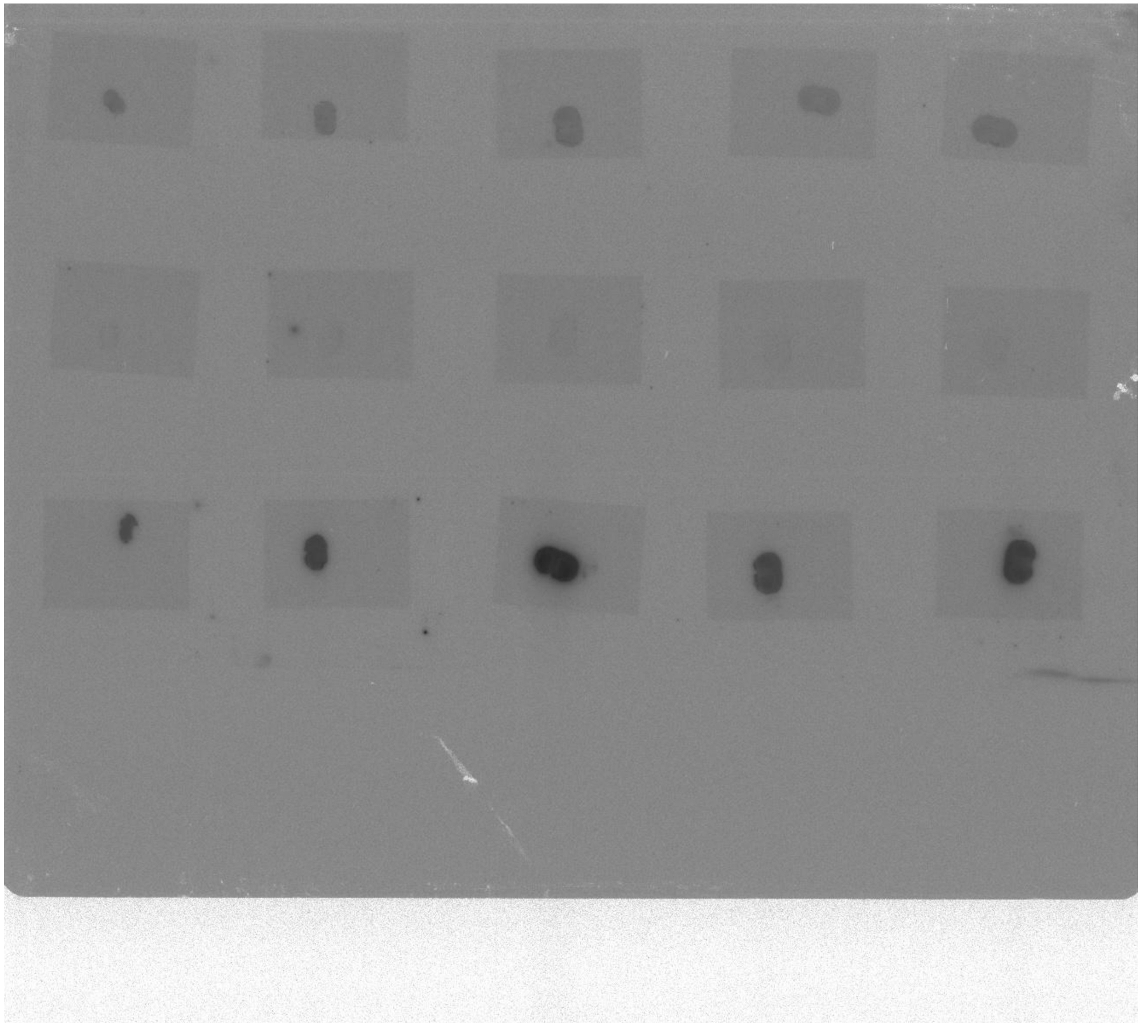

Representative autoradiograms of coronal brain sections obtained at 6 h, 12 h, and 24 h after administration of [^{125}I]**1c**. Rows correspond to different time points (from top to bottom: 12 h, 24 h, and 6 h). Columns correspond to different coronal levels (from left to right: bregma A1.0 mm, 0.0 mm, P1.4 mm, P2.0 mm, and P2.1–2.2 mm). Images are shown prior to trimming.
